# Transcriptome-Informed Digital Twins Reveal a Functional Response Landscape in Prostate Cancer

**DOI:** 10.3390/ijms272110053

**Published:** 2026-11-09

**Authors:** Radosław Dzik, Joanna Chwał, Ewaryst J. Tkacz

**Affiliations:** 1 Department of Clinical Engineering, Academy of Silesia, ul. Rolna 43, 40-555 Katowice, Poland; joanna.chwal@akademiaslaska.pl (J.C.); ewaryst.tkacz@wst.pl (E.J.T.); 2 Department of Respiratory Medicine and Infectious Diseases, Biomedical Research in Endstage and Obstructive Lung Disease Hannover BREATH, Hannover Medical School, Carl-Neuberg-Str. 1, 30625 Hannover, Germany

**Keywords:** prostate cancer, digital twin, computational modelling, transcriptomics, pathway perturbation, functional response landscape

## Abstract

Prostate cancer (PCa) shows substantial inter-sample molecular heterogeneity, yet static molecular profiles provide limited information about how individual tumours respond to changes in signalling activity. We developed a transcriptome-informed digital-twin framework that translates tumour-specific molecular states into simulated functional responses. RNA-sequencing data from 492 primary TCGA-PRAD tumour samples were scored across 1628 Reactome pathways. Of these, 220 unique pathways (232 pathway-to-node assignments) were selected through biologically informed mapping and aggregated into eight functional nodes used to personalise individual digital twins (DTs). Each DT was simulated under six conditions: baseline and five perturbations involving AR (androgen receptor) suppression, PI3K/AKT suppression, TGF-β induction, combined AR and PI3K/AKT suppression, and combined AR suppression with TGF-β induction. These simulations produced a 42-feature behavioural fingerprint for each tumour. PCA identified a continuous functional response space, with the first three components accounting for 80.75% of behavioural variance. The dominant response axis was associated with Gleason score (Spearman ρ=0.30, FDR-adjusted p<0.001), while TP53 mutation status was associated with PC1 and PC3 after multiple-testing correction. Responses to individual perturbations varied between tumour samples and were coordinated across pathway interventions. Combined perturbations were largely additive, with relative deviations below 3% across the principal functional outputs. The dominant simulation-derived axis closely corresponded to the major static transcriptomic axis, indicating that the model primarily re-expressed, rather than replaced, the underlying molecular structure. The major variance structure was preserved across personalisation strengths, whereas discrete HDBSCAN solutions depended on the parameter setting. Ablation analysis showed that transcriptome-informed parameter personalisation was necessary to preserve inter-sample differences in baseline steady states. Overall, the results describe PCa heterogeneity as a continuous functional response space and position transcriptome-informed DTs as a mechanistic re-expression of molecular profiles that enables explicit, hypothesis-generating counterfactual perturbation analyses.

## 1. Introduction

In the United States, prostate cancer remains one of the most commonly diagnosed malignancies in men, with an estimated 333,830 new cases and 36,320 deaths projected for 2026 [1]. Although many PCa tumours grow slowly and may remain clinically silent for years, others become aggressive, progress rapidly, and eventually become resistant to all available therapies [2].

The heterogeneity of prostate cancer is known to be due to biological differences between tumours. Therefore, a given prostate cancer may harbour different genetic and/or epigenetic changes, and even within a given tumour, there can be significant variation between different areas [3]. Hence, two patients with similar appearances of prostate cancer may have very different clinical courses and respond differently to treatment [4]. Although molecular characterisation of a tumour can reveal some of this heterogeneity, the critical issue of how a given set of molecular characteristics enable a tumour to behave as a tumour, particularly when signalling pathways are disrupted by cancer or by treatment, remains generally unresolved. The Cancer Genome Atlas (TCGA) provided the molecular characterisation of primary prostate cancer and identified several molecular subtypes of the tumour that harbour distinct sets of recurrent alterations including ETS family gene fusions and SPOP, FOXA1 and IDH1 mutations [5].

Even though molecular subtyping of PCa can uncover the biological heterogeneity between the different tumour types, conversion of corresponding high-dimensional cancer data into behavioural models is not without big challenges. The static molecular profiles which are typically used for subtyping of cancer provide little information about the behaviour of individual tumours upon perturbation of certain signalling pathways. Functional modelling of cancer can, however, complement these molecular subtypes by describing how patient-specific molecular states change upon perturbation of defined signalling pathways and how tumours of patients with different molecular states react differently to such perturbations.

The term digital twin remains heterogeneously defined in healthcare, particularly with respect to whether continuous or real-time updating is an essential requirement [6,7,8]. In this study, we use the term in the simulation-oriented sense to denote a patient-specific computational representation in which a shared mechanistic model is personalised using molecular data from an individual tumour and can subsequently be subjected to counterfactual perturbations to simulate its functional response. Our framework does not involve continuous updating from longitudinal patient data or real-time coupling to the physical patient and should therefore be distinguished from dynamically updated or monitoring digital twins. Rather, each digital twin represents a data-informed computational instance of the same dynamical system, parameterised by the transcriptomic state of an individual tumour and used to investigate how inter-sample molecular heterogeneity may translate into differences in simulated response behaviour.

To investigate this possibility, we developed a transcriptome-informed digital-twin framework based on TCGA-PRAD data to characterise patient-specific functional response behaviour in prostate cancer. Transcriptome-derived pathway activities were integrated into an eight-node dynamical model and used to personalise both the initial states and production parameters of individual digital twins. Each model was subsequently subjected to a defined panel of in silico pathway perturbations targeting AR (androgen receptor), PI3K/AKT and TGF-β signalling, individually and in combination, to generate multidimensional behavioural fingerprints. We then examined whether these simulated responses formed a continuous functional response landscape and characterised its relationship with the molecular inputs of the model. Finally, we assessed associations between the principal response axes and Gleason score, as well as PTEN, TP53 and SPOP mutation status, and evaluated the robustness of the response structure to model personalisation strength. This framework was designed as a mechanistic and hypothesis-generating approach for translating static tumour transcriptomic profiles into patient-specific functional response representations rather than as a clinically validated predictive model.

## 2. Results

### 2.1. Construction of Transcriptome-Informed Patient-Specific Digital Twins

**Figure 1:**
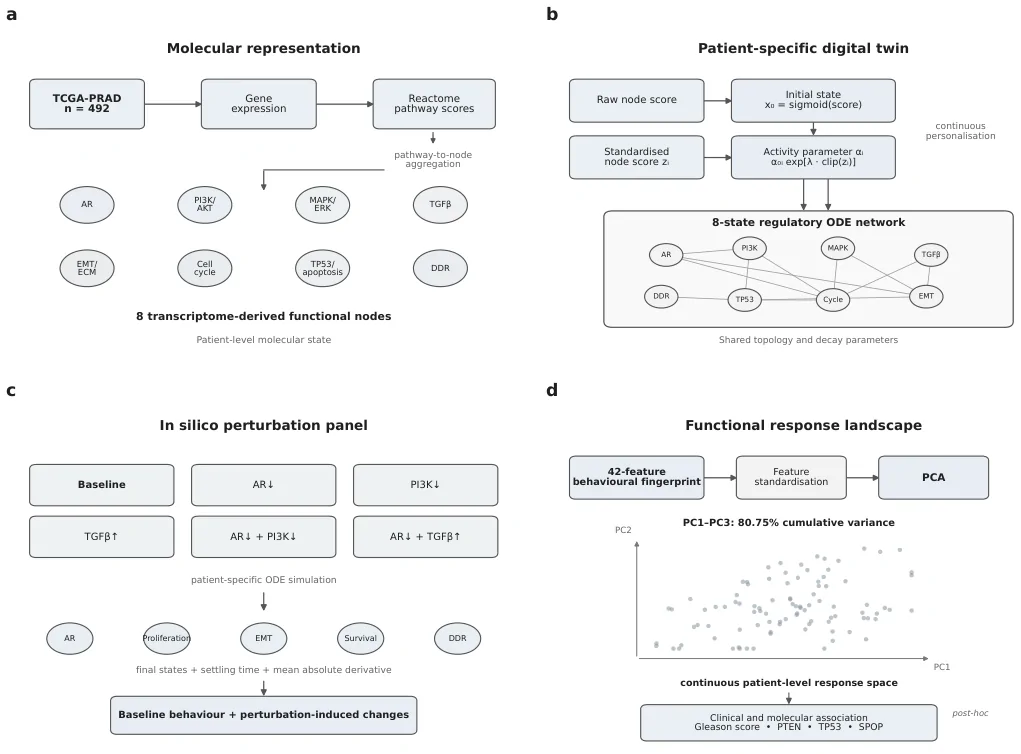
Transcriptome-informed digital-twin framework for patient-specific functional response modelling in prostate cancer. (**a**) TCGA-PRAD transcriptomic data from 492 tumours were transformed into Reactome pathway activity scores and aggregated into eight functional model nodes representing the AR programme, PI3K/AKT, MAPK/ERK, TGF-β, EMT/ECM/invasion, cell-cycle/proliferation, TP53/apoptosis, and DNA-damage response (DDR). (**b**) Raw node scores defined patient-specific initial states, $x_{0,i}=\mathrm{sigmoid}\left(\mathrm{score}i\right)$, while cohort-standardised node scores continuously modulated basal activity parameters according to $\alpha_{i}=\alpha 0,i\exp\left[\lambda ,\mathrm{clip}\left(z_{i}\right)\right]$. Network topology and decay parameters were shared across patients. (**c**) Each digital twin was simulated under baseline conditions and five single or combined perturbations. Final functional states, settling time, and mean absolute derivative were used to construct a 42-feature behavioural fingerprint comprising baseline behaviour and perturbation-induced changes. (**d**) Standardised behavioural fingerprints were projected by principal component analysis (PCA) to define a continuous sample-level functional response landscape. Gleason score and PTEN, TP53, and SPOP mutation status were used exclusively for post-hoc clinical and molecular association analyses and did not contribute to digital-twin construction.

Transcriptomic profiles from 492 TCGA-PRAD tumours were used to construct patient-specific digital twins within a shared eight-node regulatory network (Figure 1). Gene-level expression was first transformed into Reactome pathway activity scores and subsequently aggregated into eight functional model nodes representing the AR programme, PI3K/AKT signalling, MAPK/ERK signalling, TGF-β signalling, EMT/ECM/invasion, cell-cycle/proliferation, TP53/apoptosis, and DNA-damage response (DDR). Of the 1628 Reactome pathways scored across the cohort, 220 unique pathways contributed to the eight functional nodes through 232 pathway-to-node assignments. The resulting node scores provided the molecular state representation for each tumour sample. The complete Reactome pathway-to-node mapping is provided in Supplementary Table S1.

To assess the sensitivity of the functional node definitions to pathway selection, we performed three complementary analyses. First, excluding the 12 Reactome pathways shared between functional nodes resulted in minimal changes to the original node scores (Spearman ρ=0.985–1.000 across the eight nodes). Second, because the AR node was constructed from only five pathways, we performed a leave-one-pathway-out analysis, which showed relatively stable AR scores (median Spearman ρ=0.924). Third, comparison of the Reactome-derived AR node with the independently defined MSigDB Hallmark Androgen Response signature showed moderate concordance (ρ=0.421). These analyses indicate that the node scores were relatively robust to the examined pathway-mapping modifications, although the alternative AR signature captured a partly distinct transcriptional signal.

Patient specificity was introduced at two levels. Raw transcriptome-derived node scores defined the initial state of the dynamical system, whereas cohort-standardised node scores continuously modulated the corresponding basal activity parameters ($\alpha_{i}$). All tumour samples retained the same network topology, regulatory coefficients, decay parameters, and numerical simulation settings. Somatic mutation status was not used to construct the digital twins and was retained exclusively for subsequent molecular association analyses. The 492 modelled primary-tumour samples originated from 488 unique TCGA cases, with four cases contributing two primary-tumour samples.

Each digital twin was simulated under baseline conditions and five perturbation scenarios: AR suppression, PI3K suppression, TGF-β induction, combined AR and PI3K suppression, and combined AR suppression and TGF-β induction. Five functional outputs—AR activity, proliferation, EMT, survival, and DDR—were recorded together with settling time and mean absolute trajectory derivative. Baseline behaviour and perturbation-induced changes generated 42 behavioural features per tumour sample, providing a common functional representation for downstream response-space analysis.

### 2.2. Digital Twins Form a Continuous Functional Response Landscape

**Figure 2:**
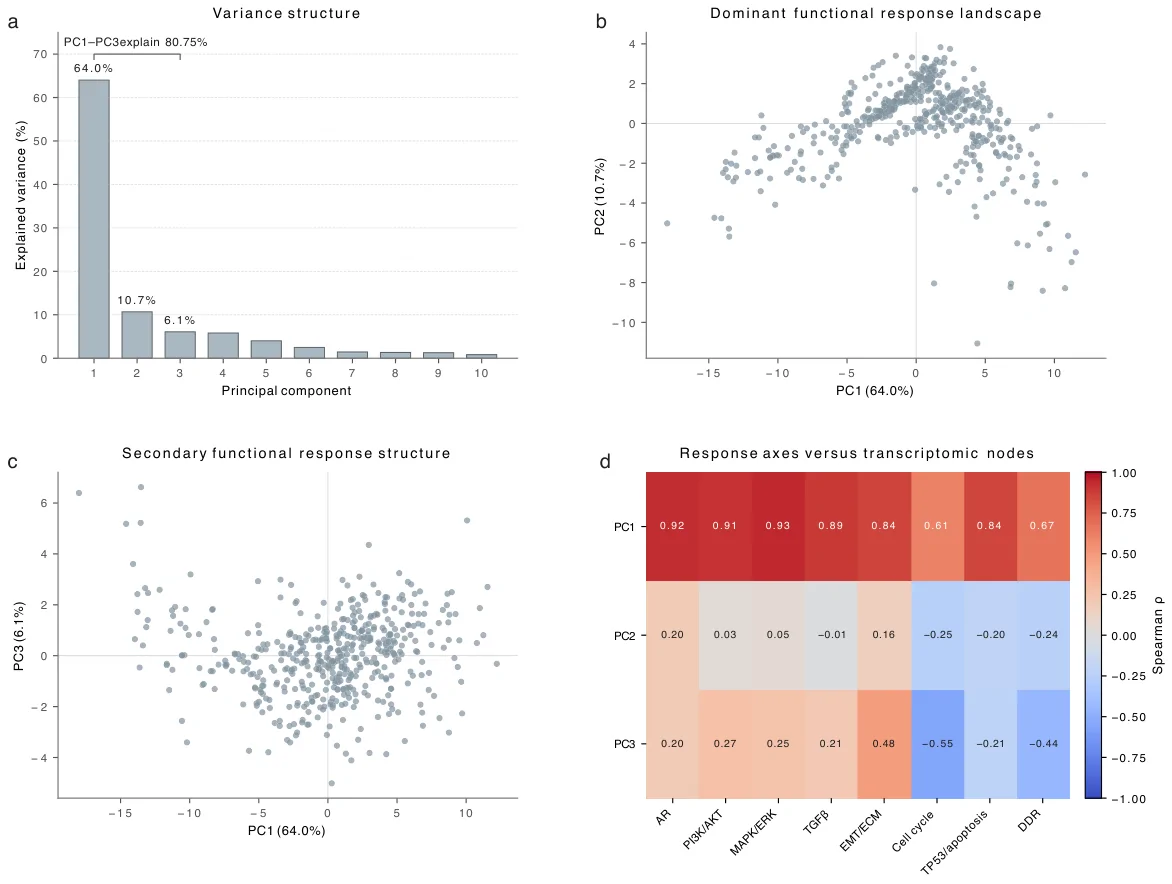
Continuous functional response landscape of transcriptome-informed prostate cancer digital twins. (**a**) Explained variance across the first 10 principal components derived from the 42-feature behavioural fingerprints. PC1, PC2, and PC3 accounted for 64.0%, 10.7%, and 6.1% of total variance, respectively, and together explained 80.75%. (**b**) Sample-level distribution in the dominant PC1-PC2 functional response space. (**c**) PC1–PC3 projection illustrating secondary functional response structure. Each point represents one of 492 patient-specific digital twins. (**d**) Spearman correlations between the first three principal-component scores and the eight transcriptome-derived functional node scores. PC1 was positively associated with activity across most nodes, consistent with a broad functional-response axis, whereas PC3 contrasted EMT/ECM activity with cell-cycle/proliferation and DNA-damage response (DDR). PCA was performed exclusively on behavioural fingerprint features; transcriptomic node scores were used in panel (**d**) for post-hoc interpretation of the resulting response axes.

Principal component analysis of the 42-feature behavioural fingerprints revealed a strongly structured but continuous functional response space (Figure 2). PC1 accounted for 64.0% of total variance, PC2 for 10.7%, and PC3 for 6.1%; together, the first three components explained 80.75% of the behavioural variance. The first 15 components accounted for 99.65% of the total variance.

The sample distribution in PC1–PC2 and PC1–PC3 space did not indicate a requirement for discrete response classes. Instead, the digital twins occupied a continuous response landscape dominated by a major PC1 axis, with additional variation represented by PC2 and PC3 (Figure 2b,c).

To determine how these functional response axes related to the transcriptomic inputs from which the digital twins were parameterised, PC coordinates were compared with the eight transcriptome-derived node scores (Figure 2d). PC1 showed strong positive correlations across most functional nodes, namely AR (ρ=0.92), PI3K/AKT (ρ=0.91), MAPK/ERK (ρ=0.93), TGF-β (ρ=0.89), EMT/ECM (ρ=0.84), TP53/apoptosis (ρ=0.84), DDR (ρ=0.67), and cell-cycle/proliferation (ρ=0.61). PC1 therefore represented a broad axis of transcriptome-informed functional activity rather than variation attributable to a single pathway. In contrast, PC2 showed substantially weaker relationships with the individual transcriptomic nodes, with the largest negative correlations observed for cell-cycle/proliferation (ρ=−0.25) and DDR (ρ=−0.24). Finally, PC3 captured a more contrasting functional pattern, correlating positively with EMT/ECM activity (ρ=0.48) while correlating negatively with cell-cycle/proliferation (ρ=−0.55) and DDR (ρ=−0.44). These relationships indicate that the dominant response axis retained substantial information from the transcriptomic inputs, whereas the secondary axes represented a different organisation of this input within the simulated behavioural space.

### 2.3. Comparison with the Static Transcriptomic Representation

To quantify the contribution of the dynamical simulation layer beyond the transcriptomic node representation, PCA was also performed directly on the eight functional-node scores. The first three static components explained 79.63%, 13.76%, and 2.17% of variance, respectively, corresponding to 95.56% cumulatively. The dominant static and simulation-derived axes were strongly related: static PC1 correlated with simulation-derived PC1 at ρ=0.976. In contrast, the correspondence between the secondary axes was weaker and involved substantial rotation of the response space. Static PC2 correlated with simulation-derived PC2 at ρ=−0.390 and with simulation-derived PC3 at ρ=−0.870, whereas static PC3 correlated with simulation-derived PC2 at ρ=0.366 and only weakly with simulation-derived PC3 (ρ=−0.132). Clinical associations were present in both representations. Static PC1 was associated with Gleason score at ρ=0.337, compared with ρ=0.300 for simulation-derived PC1. Static PC2 and PC3 were also associated with Gleason score (ρ=0.241 and −0.111, respectively), whereas the corresponding simulation-derived associations were ρ=−0.193 for both PC2 and PC3. TP53 mutation was associated with static PC1 and PC2 after FDR correction, whereas in the simulation-derived representation the strongest corrected associations involved PC1 and PC3. These comparisons show that the dominant simulation-derived axis largely preserved the broad transcriptomic gradient already present in the input node scores rather than generating an independent dominant clinical axis. The dynamical layer instead provided an explicit counterfactual representation of baseline and perturbation responses and reorganised secondary variation within that response space. Detailed numerical comparisons between the static and simulation-derived representations are provided in Supplementary Table S2.

### 2.4. Functional Response Axes Are Associated with Gleason Score and TP53 Mutation Status

**Figure 3:**
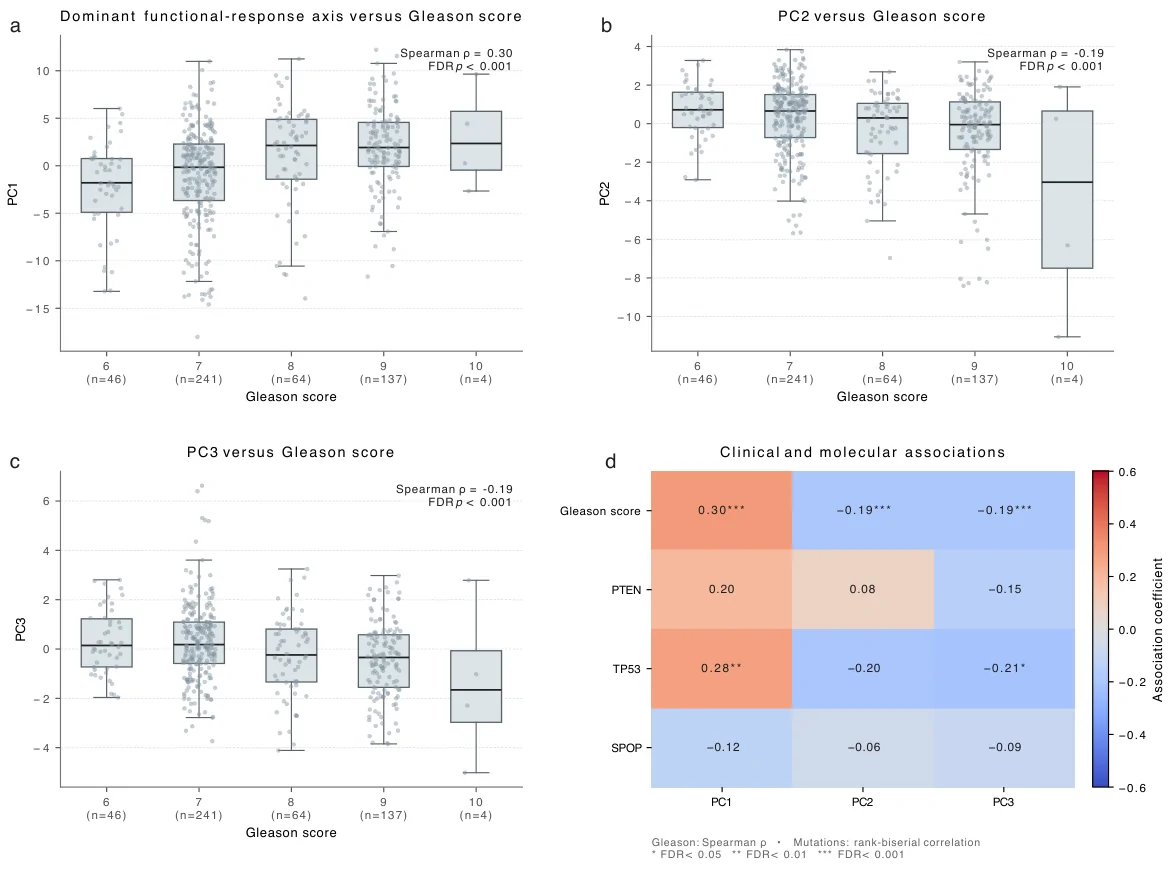
Clinical and molecular associations with the functional response landscape. (**a**–**c**) Distribution of PC1, PC2, and PC3 scores across Gleason score categories in 492 TCGA-PRAD tumours. Boxes indicate the interquartile range with the median, whiskers extend to 1.5 times the interquartile range, and individual points represent patient-specific digital twins. Sample numbers are shown below each Gleason category. Spearman correlations with Gleason score were significant for PC1 (ρ=0.30), PC2 (ρ=−0.19), and PC3 (ρ=−0.19); all remained significant after false-discovery-rate (FDR) correction (p<0.001). The Gleason 10 category contained only four tumour samples and is shown for completeness. (**d**) Associations of Gleason score and PTEN, TP53, and SPOP mutation status with the first three functional response axes. Values represent Spearman ρ for Gleason score and rank-biserial correlation coefficients for mutation status. TP53 mutation was significantly associated with PC1 and PC3 after FDR correction, whereas associations with PTEN and SPOP were not significant. Asterisks indicate FDR-adjusted significance: * p<0.05, ** p<0.01, and *** p<0.001. Clinical and mutation variables were used exclusively for post-hoc association analyses and were not included in behavioural fingerprint construction or PCA.

We next examined whether the continuous functional response landscape was associated with clinical and genomic characteristics that were not used in model construction (Figure 3). Gleason scores were available for all 492 tumour samples and ranged from 6 to 10, with 46 tumours scored as 6, 241 as 7, 64 as 8, 137 as 9, and four as 10.

PC1 showed a positive association with Gleason score (Spearman ρ=0.30, FDR-adjusted p<0.001; Figure 3a). Higher-grade tumours tended to have higher PC1 scores, placing them further along the dominant axis of functional response variation. In contrast, both PC2 and PC3 showed negative associations with Gleason score (PC2: ρ=−0.19, FDR-adjusted p<0.001; PC3: ρ=−0.19, FDR-adjusted p<0.001; Figure 3b,c). Kruskal–Wallis comparisons across Gleason-score categories were significant for all three response axes (PC1: H=44.99, FDR-adjusted $p=1.20\times 10^{-8}$, $\epsilon^{2}=0.084$; PC2: H=19.22, FDR-adjusted $p=7.12\times 10^{-4}$, $\epsilon^{2}=0.031$; PC3: H=20.06, FDR-adjusted $p=7.12\times 10^{-4}$, $\epsilon^{2}=0.033$), with the largest grade-related effect observed for PC1.

Mutation associations were examined separately for PTEN, TP53, and SPOP (Figure 3d). PTEN, TP53, and SPOP mutations were present in 16, 54, and 55 of the 492 tumour samples, respectively. Among these genes, TP53 showed the clearest relationship with the functional response space. TP53-mutant tumours had higher PC1 scores (rank-biserial correlation $r_{\mathrm{rb}}=0.28$, FDR-adjusted p=0.0067) and lower PC3 scores ($r_{\mathrm{rb}}=-0.21$, FDR-adjusted p=0.045). A similar negative association was observed for PC2, although it did not remain significant after correction for multiple testing ($r_{\mathrm{rb}}=-0.20$, FDR-adjusted p=0.051). Neither PTEN nor SPOP coding mutation status was significantly associated with any of the three principal response axes after FDR correction. Because PTEN coding mutation status does not capture copy-number loss, PTEN copy-number associations were examined separately in the subsequent analysis. Thus, the continuous functional response space was related to tumour grade and, more specifically, to TP53 mutation status, even though neither clinical grade nor mutation status was used to construct the digital twins.

### 2.5. Tumour Purity Contributes to, but Does Not Explain, the Principal Response Associations

Tumour purity was associated with several transcriptome-derived functional nodes and with the dominant response-space axes. Using Consensus Purity Estimate (CPE), available for 487 of the 492 tumour samples, purity correlated negatively with AR (ρ=−0.35), PI3K/AKT (ρ=−0.42), MAPK/ERK (ρ=−0.43), TGF-β (ρ=−0.38), EMT/ECM (ρ=−0.54), TP53/apoptosis (ρ=−0.26), and DDR (ρ=−0.10), whereas the association with cell-cycle/proliferation was comparatively weak. CPE was also associated with static PC1 (ρ=−0.41) and simulation-derived PC1 (ρ=−0.44). Simulation-derived PC3 was negatively associated with purity (ρ=−0.37), whereas PC2 showed little relationship with CPE. Adjustment for CPE attenuated but did not remove the association between Gleason score and the simulation-derived response axes. The partial Spearman correlations were ρ=0.232 for PC1, ρ=−0.185 for PC2, and ρ=−0.285 for PC3, with all three remaining significant after FDR correction. TP53 associations likewise persisted after purity adjustment, with adjusted effects observed for PC1, PC2, and PC3. The high correlations among the transcriptome-derived nodes were also only modestly reduced after adjustment for CPE: the median pairwise node correlation decreased from approximately 0.746 to 0.722. An analysis using ESTIMATE-derived purity produced the same overall interpretation. These results indicate that tumour purity contributed to the global transcriptomic and response-space structure but did not account fully for either the covariance among functional nodes or the observed Gleason and TP53 associations. Detailed tumour-purity associations and purity-adjusted clinical and molecular results are reported in Supplementary Table S3.

### 2.6. PTEN Copy-Number Loss Is Associated with Digital-Twin Behavioural Variation

Somatic mutations in PTEN, TP53, and SPOP were identified in 16 (3.3%), 54 (11.0%), and 55 (11.2%) of the 492 modelled tumour samples, respectively.

The absence of a significant association for PTEN coding mutation was examined further using post hoc copy-number data, because mutation status alone captures only one form of PTEN alteration. DNAcopy-derived PTEN copy-number estimates were available for 489 of the 492 modelled tumour samples. Continuous PTEN copy number was negatively associated with PC1 (Spearman’s ρ=−0.238, FDR-adjusted $p=3.18\times 10^{-7}$), whereas associations with PC2 (ρ=0.082) and PC3 (ρ=0.021) were not significant. These principal components were derived from 42 digital-twin behavioural features encompassing baseline model characteristics and simulated perturbation responses.

The PC1 association was also observed across multiple threshold-based definitions of PTEN deletion. Depending on the threshold applied to the copy-number segment value (−0.2 to −0.5), 69–139 samples were classified as deletion positive, and deletion-positive tumours showed consistently higher PC1 scores. For example, at a threshold of −0.3, 114 samples were classified as PTEN-deleted, with a PC1 effect size of $r_{\mathrm{rb}}=0.225$ (FDR-adjusted p<0.001). Associations involving PC2 and PC3 varied across deletion thresholds.

When PTEN mutation and copy-number loss were considered jointly at the −0.3 threshold, 119 of 489 tumours (24.3%) were classified as PTEN-altered. Combined alteration status was significantly associated with PC1 ($r_{\mathrm{rb}}=0.219$, FDR-adjusted $p=9.76\times 10^{-4}$), PC2 ($r_{\mathrm{rb}}=-0.143$, FDR-adjusted p=0.0189), and PC3 ($r_{\mathrm{rb}}=-0.183$, FDR-adjusted p=0.00404).

Thus, the earlier null result for PTEN specifically concerned coding mutation status and did not imply the absence of a relationship between broader PTEN alterations and digital-twin behavioural variation. Copy-number loss, evaluated independently of model construction, was associated with the first principal component of the behavioural fingerprints. These exploratory associations do not establish functional PTEN inactivation. The complete PTEN mutation and copy-number association results, including deletion-threshold sensitivity analyses, are provided in Supplementary Table S6.

### 2.7. In Silico Perturbations Reveal Patient-Specific Functional Response Patterns

**Figure 4:**
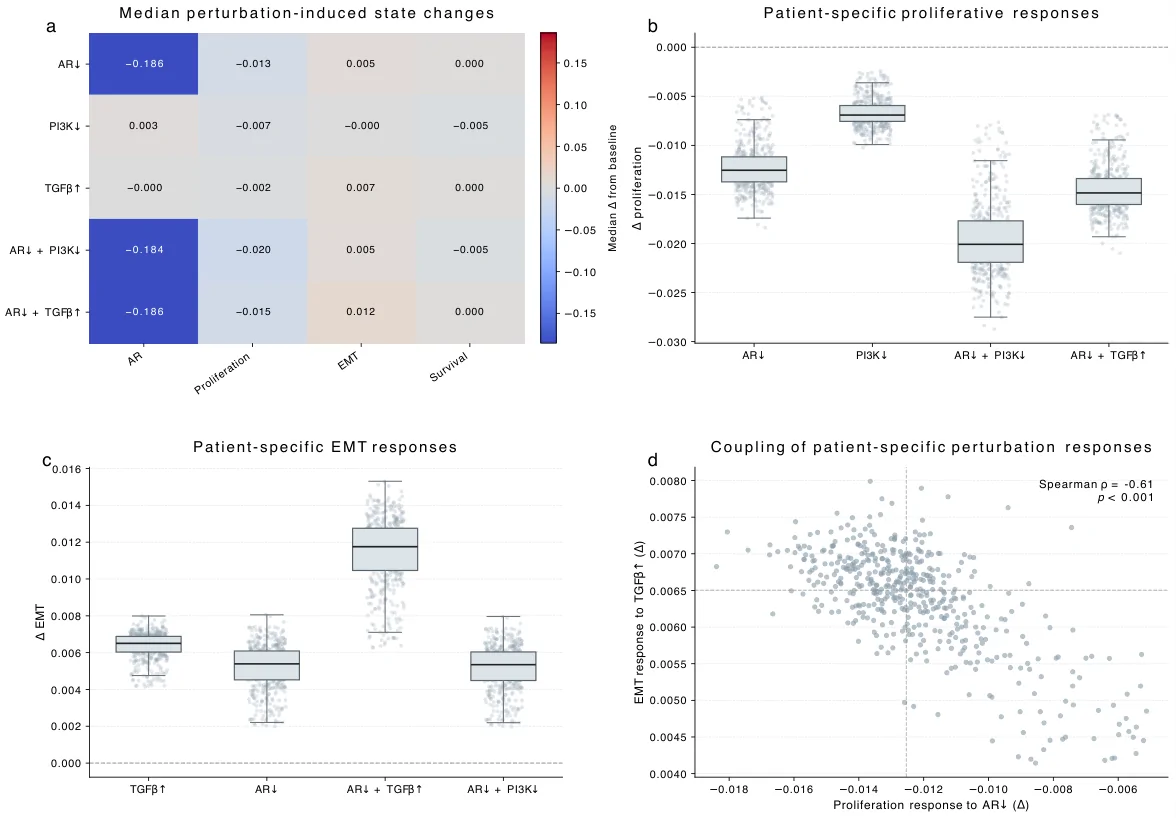
Patient-specific functional responses to in silico perturbations. (**a**) Median perturbation-induced changes from baseline across 492 patient-specific digital twins for AR activity, proliferation, EMT, and survival. Rows represent single and combined perturbations, and values denote the median change (Δ) relative to the corresponding unperturbed baseline state. DDR was omitted because perturbation-induced changes were negligible. (**b**) Patient-specific proliferative responses to AR suppression, PI3K suppression, combined AR and PI3K suppression, and combined AR suppression and TGF-β induction. (**c**) Patient-specific EMT responses to TGF-β induction, AR suppression, combined AR suppression and TGF-β induction, and combined AR and PI3K suppression. In panels (**b**,**c**), boxes indicate the interquartile range with the median, whiskers extend to 1.5 times the interquartile range, individual points represent patient-specific digital twins, and the dashed horizontal line denotes no change from baseline. (**d**) Relationship between the patient-specific proliferative response to AR suppression and EMT response to TGF-β induction. Responses were inversely associated (Spearman ρ=−0.61, p<0.001), indicating correlated variation in model-generated responses across the simulated cohort rather than a demonstrated biological interaction between the perturbations. Downward and upward arrows indicate simulated pathway suppression and activation, respectively. Dashed lines indicate no change from baseline in panels (**b**,**c**) and cohort median responses in panel (**d**).

The perturbation panel produced responses that were directionally consistent across the 492 patient-specific digital twins, although their magnitude varied considerably between tumour samples (Figure 4). AR suppression had its strongest effect on AR activity itself, with a median ΔAR of −0.186. Changes in the other functional outputs were smaller. Proliferation decreased modestly (median Δ=−0.0125), while EMT showed a slight increase (median Δ=0.0054). PI3K suppression had comparatively little effect on AR activity but reduced proliferation (Δ=−0.0069) and survival (Δ=−0.0053). TGF-β induction primarily increased EMT (Δ=0.0065), with only small median changes in the other functional outputs. Combined AR and PI3K suppression produced a median AR change of −0.184 and a median proliferation change of −0.0200, representing the largest median reduction in proliferation among the simulated perturbations. Combined AR suppression and TGF-β induction produced a similar reduction in AR activity (Δ=−0.186) together with the largest median increase in EMT (Δ=0.0118). Changes in DDR were negligible across the perturbation panel and were therefore not emphasised in the comparative response heatmap. Importantly, the distributions of sample-level responses showed that identical perturbation classes did not produce identical response magnitudes across the cohort (Figure 4b,c). This heterogeneity was evident for both proliferative responses to pathway suppression and EMT responses to TGF-β-related perturbations.

The combined perturbation responses were broadly consistent with an approximately additive pattern. For example, the median proliferation changes following individual AR and PI3K suppression were −0.0125 and −0.0069, respectively, compared with −0.0200 under combined suppression. However, these cohort-level median responses do not constitute a formal patient-level additivity test. Given the deterministic nature of the simulations, the biological relevance of non-additive effects should be assessed primarily through their magnitude rather than statistical significance. Accordingly, the combined responses are interpreted descriptively, without inferring biological synergy or antagonism.

Responses across different perturbation mechanisms were also coupled. The proliferative response to AR suppression was inversely associated with the EMT response to TGF-β induction (Spearman ρ=−0.61, p<0.001; Figure 4d). This association was treated as a property of the simulated response landscape rather than evidence of a direct biological interaction between the two perturbations.

Because the primary perturbation magnitudes depended on the baseline state of the targeted node, the analysis was repeated using identical perturbation factors for every tumour sample, defined from the cohort medians of the primary factors (AR, 0.3451; PI3K, 0.5199; TGF-β, 1.5839). Perturbation-response features remained highly concordant with the primary model (median Spearman ρ=0.992 across response features). The AR-suppression proliferation versus TGF-β-induction EMT association also persisted and became slightly stronger (ρ=−0.679). PC1 and PC2 were highly concordant between the primary and uniform-perturbation models (ρ=0.996 and 0.973, respectively), whereas PC3 was less stable (ρ=−0.250). Thus, the dominant response structure and cross-perturbation coupling were not explained by the state-dependent intervention magnitude, although the third response axis was sensitive to this modelling choice. The complete comparison between patient-dependent and uniform perturbation settings is provided in Supplementary Table S4.

**Figure A1:**
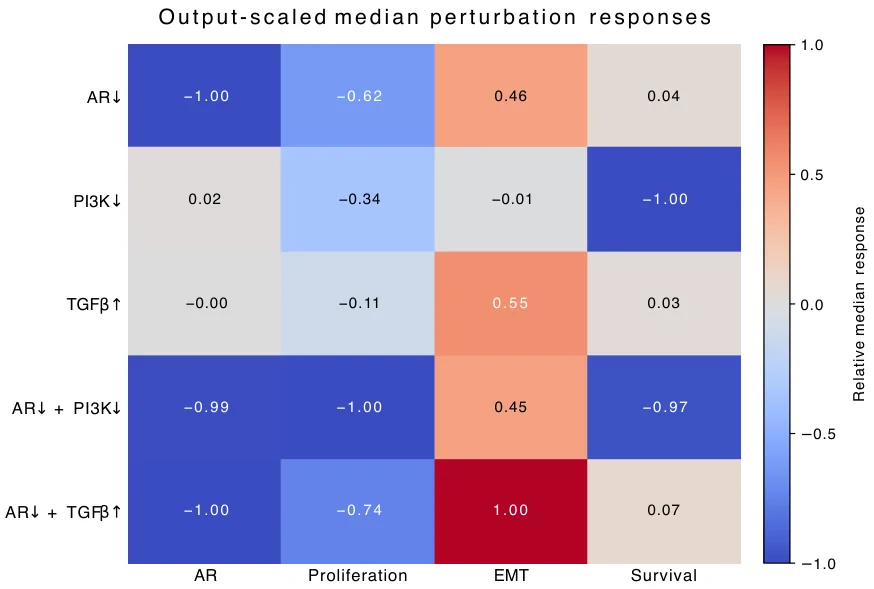
Output-scaled visualisation of median perturbation-induced functional responses. Median changes from baseline across 492 TCGA-PRAD tumour samples are shown for AR activity, proliferation, EMT, and survival under the five simulated perturbation conditions. To facilitate comparison of response patterns across outputs with markedly different absolute effect magnitudes, each output column was scaled independently by its maximum absolute median response across the five perturbation conditions. Scaled values therefore range from −1 to +1 within each functional output, with the sign preserving the direction of the original response and the absolute value representing its magnitude relative to the largest median response observed for that output. This scaling was applied only for visualisation; all quantitative analyses and reported effect estimates were based on the original unscaled perturbation responses shown in Figure 4. DDR was omitted because its perturbation-induced changes were numerically close to zero, making relative scaling uninformative.

Because the absolute response magnitudes differed markedly among outputs, particularly between AR and the other functional states, an output-scaled version of the median-response heatmap was additionally generated to facilitate comparison of relative response patterns (Figure A1). Each output was scaled independently to its maximum absolute median response across perturbation conditions. This transformation was used only for visualisation; all quantitative analyses were based on the original unscaled responses.

### 2.8. Model Responses Are Robust to Moderate Parameter Perturbation

The numerical parameterisation of the shared dynamical network was examined by globally scaling either all non-zero interaction weights or all reference production parameters by ±20%. The principal perturbation responses were highly conserved under these changes. Median rank correlations of perturbation-response features with the primary model were 0.9999 for both 0.8× and 1.2× interaction-weight scaling, 0.9986 for $0.8\times \alpha_{0}$, and 0.9977 for $1.2\times \alpha_{0}$. The dominant PCA axis was similarly stable, with PC1 correlations of 0.995–0.999 relative to the primary model. PC1 explained 63.7–64.5% of variance after interaction-weight scaling and 60.3–63.7% after reference-production scaling, compared with 64.0% in the primary model. Secondary axes were more sensitive, particularly to changes in $\alpha_{0}$, indicating rotation of the lower-variance response structure. The inverse association between the proliferative response to AR suppression and the EMT response to TGF-β induction was preserved in every parameter scenario (ρ=−0.44 to −0.68). The direction of the principal Gleason and TP53 associations with PC1 was also retained. These results support robustness of the dominant response structure to moderate global changes in the selected numerical parameters, while indicating greater parameter sensitivity of the secondary response axes. The analysis represents local sensitivity around the selected model rather than formal parameter-identifiability or global uncertainty analysis. Detailed results of the model-parameter sensitivity analyses are provided in Supplementary Table S5a.

### 2.9. Functional-Node Construction Is Robust to Pathway Overlap and Alternative AR Definitions

Of the 220 unique Reactome pathways included in the node definitions, 12 were assigned to two functional nodes, resulting in 232 pathway-to-node assignments. Excluding all 12 shared pathways had little effect on the resulting node scores. Spearman correlations between the primary and overlap-excluded definitions ranged from ρ=0.985 for TP53/apoptosis to ρ=1.000 for AR, PI3K/AKT, and TGF-β, with similarly high concordance for MAPK/ERK (ρ=0.998), EMT/ECM (ρ=0.992), cell-cycle/proliferation (ρ=0.999), and DDR (ρ=0.997). Because only five Reactome pathways contributed to the AR-programme node, its dependence on individual pathways was examined separately. Leave-one-pathway-out AR scores remained strongly correlated with the primary AR node, with correlations ranging from ρ=0.906 to 0.935 (median ρ=0.924). Thus, no single constituent Reactome pathway determined the AR composite. An independently defined Hallmark androgen-response score provided a further benchmark of the AR-node definition. Of the 101 genes in HALLMARK_ANDROGEN_RESPONSE, 100 were represented in the filtered TCGA-PRAD expression matrix. The Hallmark score correlated most strongly with the Reactome-derived AR-programme node (ρ=0.421, FDR-adjusted $p=1.19\times 10^{-21}$) among the eight functional nodes. The individual AR-related Reactome pathways nevertheless showed heterogeneous relationships with the Hallmark score, consistent with the broader pathway-level composition of the AR-programme node. These analyses support robustness of the selected AR representation without treating the Hallmark comparison, which used the same TCGA expression cohort, as independent validation. Detailed pathway-mapping robustness results, including shared-pathway exclusion and alternative AR definitions, are provided in Supplementary Table S5b.

### 2.10. Response-Space Variance Is Preserved Across Personalisation Strengths, Whereas Discrete Clustering Is Parameter Sensitive

**Figure 5:**
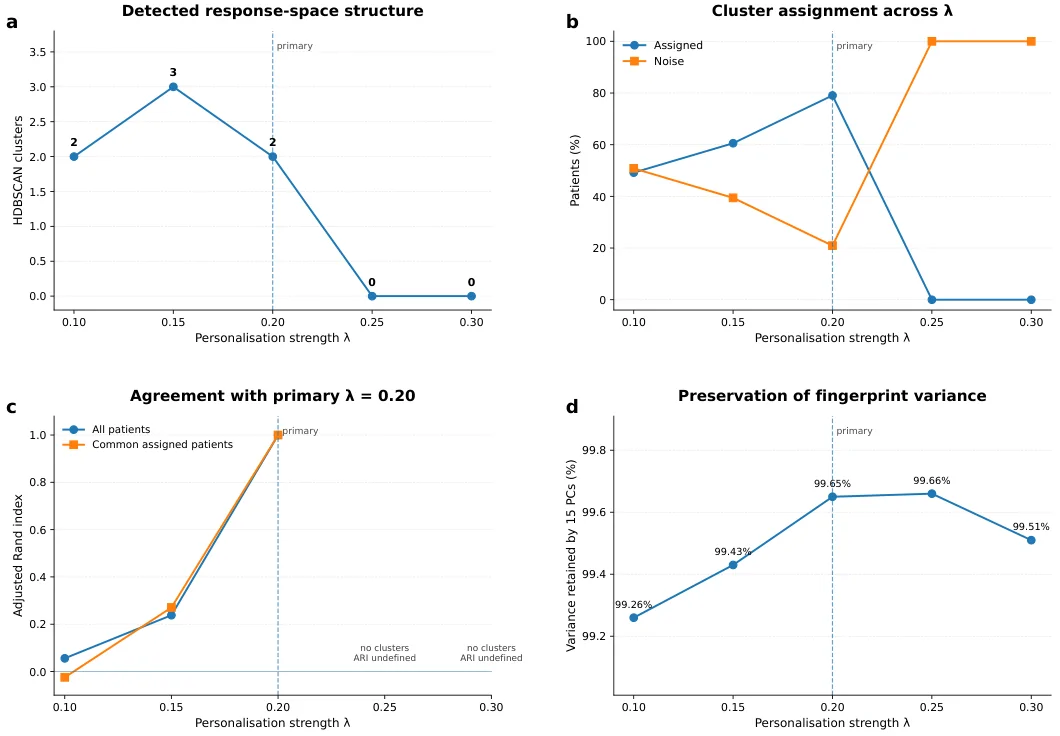
Sensitivity of response-space structure to transcriptome-informed personalisation strength. The strength of basal-activity parameter personalisation (λ) was varied from 0.10 to 0.30, with λ=0.20 representing the primary model setting (vertical dashed line). (**a**) Number of response-space clusters detected by HDBSCAN at each value of λ. Two clusters were detected at λ=0.10, three at λ=0.15, and two at λ=0.20, whereas no clusters were detected at λ=0.25 or 0.30. (**b**) Proportion of tumour samples assigned to an HDBSCAN cluster or classified as noise across personalisation strengths. At the primary setting, 79.1% of samples were assigned and 20.9% were classified as noise. (**c**) Agreement of cluster assignments with the primary λ=0.20 solution, quantified using the adjusted Rand index (ARI) for all samples and for samples assigned to clusters in both compared solutions. ARI was undefined at λ=0.25 and 0.30 because HDBSCAN detected no clusters. (**d**) Cumulative behavioural-fingerprint variance retained by the first 15 principal components across λ, ranging from 99.26% to 99.66%. Thus, the major variance structure of the functional response space was preserved across the tested personalisation strengths, whereas its discretisation into HDBSCAN clusters was strongly parameter dependent. HDBSCAN clustering was therefore treated as an exploratory analysis rather than evidence for stable discrete functional subtypes.

The strength of transcriptome-informed parameter personalisation was evaluated across λ=0.10, 0.15, 0.20, 0.25, and 0.30, with λ=0.20 defined a priori as the primary model setting (Figure 5). The overall dimensional structure of the behavioural fingerprints remained highly concentrated across the complete sensitivity range. The first 15 principal components explained 99.26%, 99.43%, 99.65%, 99.66%, and 99.51% of variance for λ=0.10, 0.15, 0.20, 0.25, and 0.30, respectively.

In contrast, discrete HDBSCAN assignments were sensitive to the personalisation strength. Two clusters were detected at λ=0.10, three at λ=0.15, and two at the primary value of λ=0.20. At λ=0.25 and 0.30, HDBSCAN did not identify discrete clusters and classified the response space as noise. At λ=0.20, 389 of 492 tumour samples (79.1%) received a cluster assignment and 103 (20.9%) were classified as noise, whereas the assigned fraction was lower at λ=0.10 and 0.15. Because no clusters were detected at λ=0.25 or 0.30, adjusted Rand indices relative to the primary clustering were undefined for these settings rather than zero.

Agreement between the non-primary and primary cluster assignments was also limited where clusters were detected. These results indicate that the major variance structure of the functional fingerprints was retained across the tested personalisation strengths, whereas their discretisation into HDBSCAN clusters was not robust to changes in λ. Accordingly, the continuous PCA-derived response landscape was retained as the primary representation of inter-sample functional heterogeneity, while clustering was treated as an exploratory property of the response space rather than evidence for stable discrete molecular or functional subtypes.

### 2.11. Combined Perturbations Are Predominantly Additive

**Figure 6:**
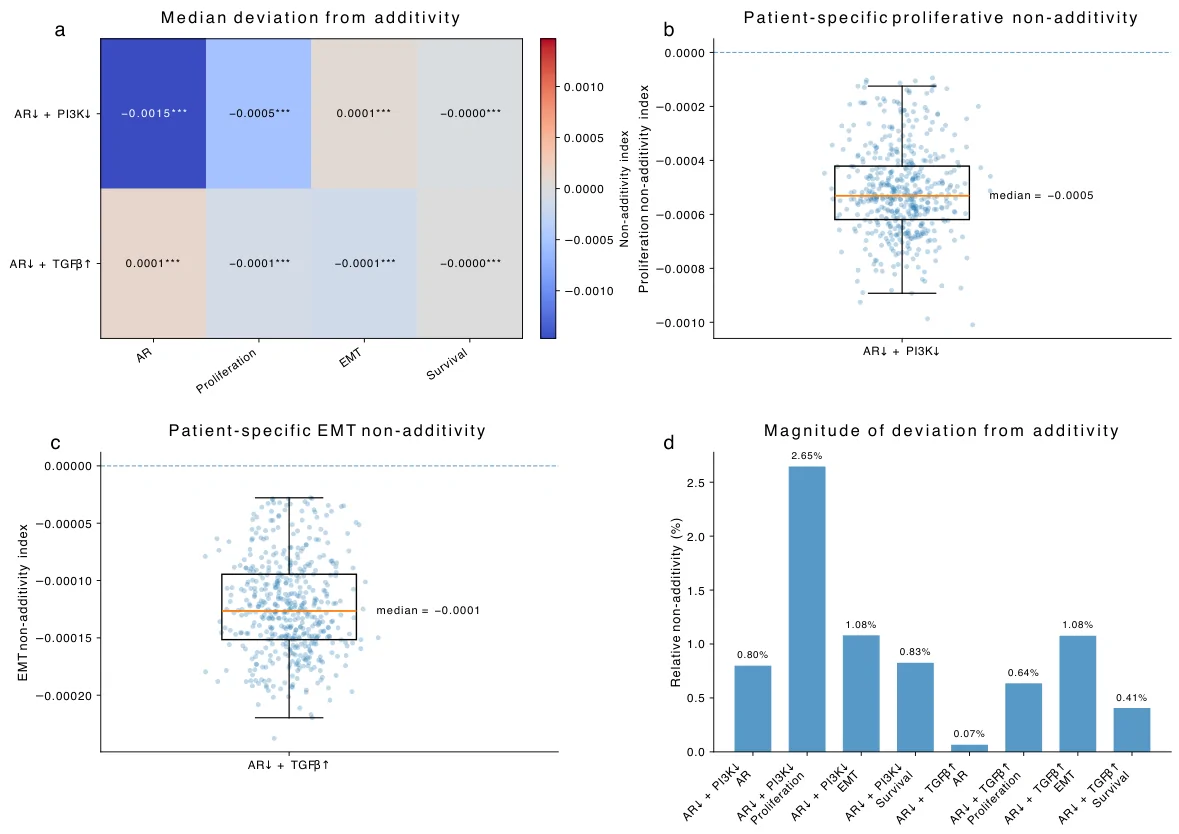
Non-additivity of combined perturbation responses in patient-specific digital twins. (**a**) Median deviation from additivity for combined AR and PI3K suppression (AR↓ + PI3K↓) and combined AR suppression and TGF-β induction (AR↓ + TGFβ↑). The non-additivity index was defined as the observed response to the combined perturbation minus the sum of the responses to the corresponding single perturbations. Positive and negative values therefore indicate deviations above and below the additive expectation, respectively. Asterisks indicate FDR-adjusted significance: *** p<0.001. (**b**) Patient-specific proliferative non-additivity for combined AR and PI3K suppression. (**c**) Patient-specific EMT non-additivity for combined AR suppression and TGF-β induction. In panels (**b**,**c**), individual points represent patient-specific digital twins, boxes indicate the interquartile range with the median, whiskers extend to 1.5 times the interquartile range, and the dashed horizontal line denotes exact additivity. (**d**) Magnitude of the median deviation from additivity expressed relative to the corresponding observed combination response. Relative deviations were small across functional outputs, with the largest value of 2.65% observed for the proliferative response to combined AR and PI3K suppression. DDR was excluded from the relative comparison because the corresponding perturbation-induced changes were close to zero, making ratio-based estimates unstable. Overall, the small effect sizes indicate that combination responses generated by the model were predominantly additive despite statistically consistent deviations from exact additivity.

To assess whether the two combination perturbations generated responses beyond those expected from their constituent single perturbations, a non-additivity index was calculated as the observed combination effect minus the sum of the corresponding single-perturbation effects (Figure 6). The resulting deviations from exact additivity were evaluated primarily in terms of their absolute and relative magnitudes. Because the simulations were deterministic, statistical significance across the 492 tumour samples does not quantify uncertainty in model parameters or experimental measurements. We therefore emphasised effect sizes rather than *p*-values when interpreting the combined perturbation responses.

For combined AR and PI3K suppression, the median non-additivity indices were approximately −0.00147 for AR activity, −0.00053 for proliferation, +0.00006 for EMT, and −0.00004 for survival. Relative to the magnitude of the corresponding observed combination responses, these deviations represented approximately 0.80%, 2.65%, 1.08%, and 0.83%, respectively. The largest relative departure from additivity was therefore observed for the proliferative response to combined AR and PI3K suppression, but remained below 3%.

For combined AR suppression and TGF-β induction, the corresponding median deviations were approximately +0.00012 for AR activity, −0.00009 for proliferation, −0.00013 for EMT, and close to zero for survival. Relative deviations were approximately 0.07%, 0.64%, 1.08%, and 0.41%, respectively. DDR was excluded from relative non-additivity comparisons because the underlying perturbation-induced changes were close to zero, making ratio-based interpretation unstable.

The sample-level distributions confirmed that the direction of these small deviations was generally consistent across the cohort (Figure 6b,c). Nevertheless, their limited magnitude indicates that the combination responses generated by the current network were predominantly additive under the specified model structure. These deviations were interpreted as model-level non-additivity and not as evidence of pharmacological synergy or antagonism.

### 2.12. Parameter Personalisation Prevents Collapse to a Common Steady State

**Figure 7:**
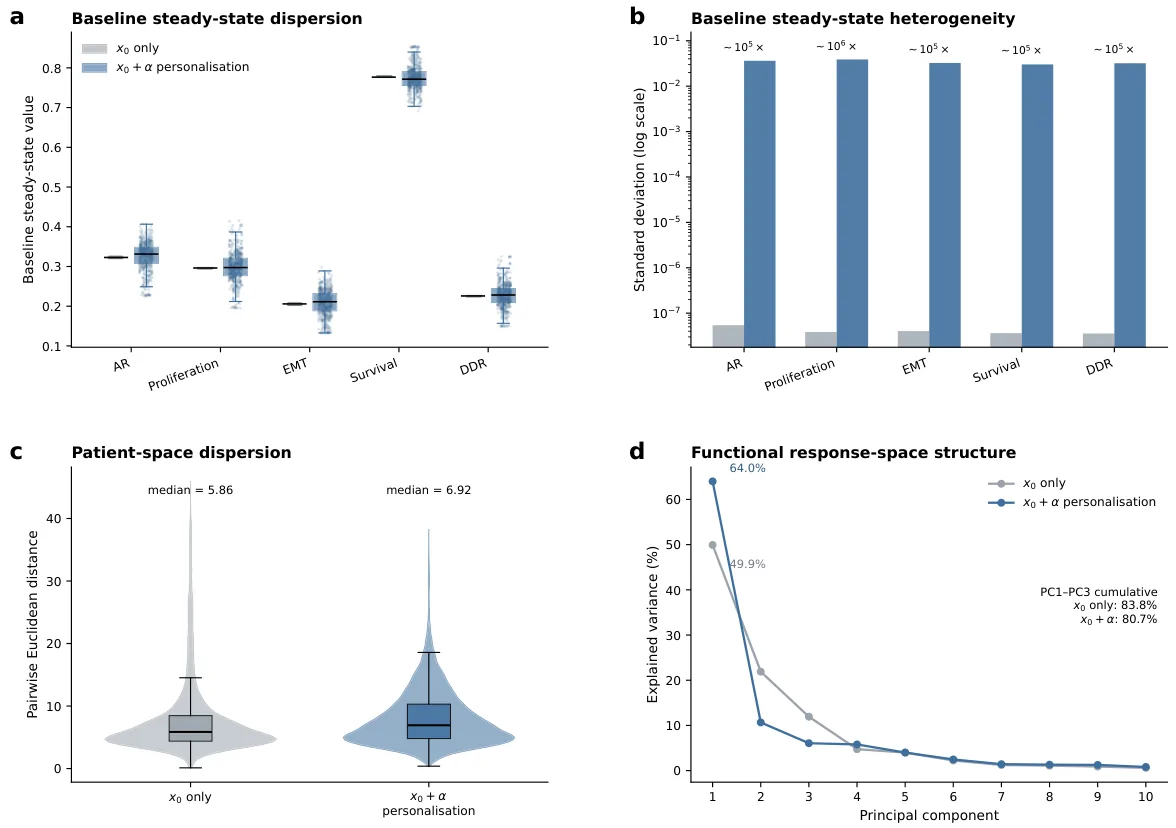
Effect of transcriptome-informed parameter personalisation on inter-sample functional heterogeneity. (**a**) Baseline steady-state values of AR activity, proliferation, EMT, survival, and DNA-damage response (DDR) in the $x_{0}$-only model and in the primary $x_{0}+\alpha$ personalisation model. In the $x_{0}$-only configuration, transcriptome-derived node scores defined patient-specific initial states, whereas basal activity parameters were shared across patients. The primary model additionally used cohort-standardised transcriptomic node scores to personalise the corresponding basal activity parameters ($\alpha_{i}$). Individual points represent patient-specific digital twins. (**b**) Between-sample standard deviations of the five baseline steady-state outputs shown on a logarithmic scale. Addition of α personalisation increased steady-state heterogeneity by approximately five to six orders of magnitude relative to the $x_{0}$-only model. (**c**) Distribution of pairwise Euclidean distances between the 42-feature behavioural fingerprints. Median pairwise distance increased from 5.86 in the $x_{0}$-only model to 6.92 following α personalisation. Boxes indicate the interquartile range with the median, and whiskers extend to 1.5 times the interquartile range. (**d**) Explained variance across the first 10 principal components for the two parameterisation strategies. PC1 accounted for 49.9% of variance in the $x_{0}$-only model and 64.0% in the $x_{0}+\alpha$ model, while the cumulative variance explained by PC1–PC3 was 83.8% and 80.7%, respectively. The ablation demonstrates that transcriptome-derived initial conditions alone were insufficient to preserve inter-sample differences at baseline steady state under the shared dynamical parameterisation, whereas continuous transcriptome-informed parameter personalisation maintained patient-specific functional heterogeneity and reorganised the behavioural response space.

Finally, we performed a mechanistic ablation to determine whether transcriptome-derived initial conditions alone were sufficient to maintain patient-specific functional heterogeneity (Figure 7). In the $x_{0}$-only model, each tumour sample retained a transcriptome-derived initial state, but all basal activity parameters were shared. Under this configuration, baseline simulations converged towards effectively identical steady states across the 492 samples. The between-sample standard deviations of the five baseline functional outputs were only $3.6\times 10^{-8}$ to $5.4\times 10^{-8}$, indicating near-complete loss of initial-state heterogeneity at steady state. This convergence is an expected consequence of applying a common dynamical parameterisation after allowing the sample-specific initial states to evolve over the same autonomous system.

In contrast, the primary model combining transcriptome-derived initial conditions with transcriptome-informed α personalisation retained substantial between-sample differences in the final functional states. Standard deviations ranged from approximately 0.030 to 0.039 across AR activity, proliferation, EMT, survival, and DDR, corresponding to increases of approximately five to six orders of magnitude compared with the $x_{0}$-only model (Figure 7a,b).

Parameter personalisation also reorganised the sample-level behavioural response space. The median pairwise Euclidean distance between tumour-sample fingerprints increased from 5.86 in the $x_{0}$-only model to 6.92 in the primary model, although the $x_{0}$-only configuration showed a longer upper tail of pairwise distances (Figure 7c). The variance structure was likewise redistributed: PC1 accounted for 49.9% of variance in the $x_{0}$-only model compared with 64.0% after α personalisation, whereas the cumulative contribution of PC1–PC3 remained broadly comparable (83.8% and 80.7%, respectively; Figure 7d).

These ablation results show that, under the shared parameterisation used here, patient-specific initial conditions alone preserved transient differences but were insufficient to maintain inter-sample heterogeneity in baseline steady states. Continuous transcriptome-informed modulation of basal activity parameters therefore provided the persistent sample-specific functional variation used in the primary digital-twin analysis.

### 2.13. Comparison of Simulated AR-Suppression Responses with Independent Experimental Data

The direction of the simulated AR-suppression responses was compared with two independent, publicly available transcriptomic datasets from enzalutamide-treated LNCaP prostate cancer cells: GSE220097 [9] and GSE202755 [10]. In GSE220097, comparison of R1881-stimulated cells with cells treated with R1881 plus enzalutamide for 22 h showed a marked reduction in the Hallmark Androgen Response score (Δ=−1.568, $r_{\mathrm{rb}}=-1.00$, p<0.001). The E2F Targets score, used as an experimental proxy for proliferation-related transcriptional activity, also decreased (Δ=−0.513, $r_{\mathrm{rb}}=-1.00$, p<0.001). Both changes were directionally consistent with the simulated reductions in AR activity and proliferation following AR suppression.

In GSE202755, DHT-stimulated LNCaP cells were compared with cells treated with DHT plus enzalutamide for 72 h. Despite the limited sample size (three samples per condition), both the Hallmark Androgen Response and E2F Targets scores decreased consistently in the treated group ($r_{\mathrm{rb}}=-1.00$ for both; Δ=−0.434 and −1.004, respectively). The minimum attainable exact two-sided Mann–Whitney *U* test *p*-value for three observations per group is 0.10; consequently, these findings were interpreted primarily in terms of response direction and effect size rather than statistical significance.

Agreement was less consistent for downstream functional programmes. EMT-related scores decreased slightly in GSE220097 but increased in GSE202755, whereas the model produced only a small positive EMT response following AR suppression. Experimental DNA-repair scores decreased in both datasets despite an approximately zero simulated DDR response. Apoptosis-related scores increased experimentally, while the simulated apoptosis response was close to zero and slightly negative. Restricting both datasets to the same represented genes did not materially change these patterns, including the positive EMT response observed in GSE202755.

Overall, the two independent enzalutamide datasets showed directional agreement with the principal simulated AR and proliferation responses, whereas correspondence for downstream programmes was incomplete. Because the experimental and simulated responses were derived from different biological systems and measured on different scales, these comparisons provide a directional experimental benchmark rather than quantitative validation of response magnitudes or patient-specific predictions. Detailed results are provided in Supplementary Table S7.

## 3. Discussion

We developed a transcriptome-informed digital-twin framework to characterise functional heterogeneity in prostate cancer through simulated responses to defined pathway perturbations. The results indicate that this heterogeneity is better represented as a continuous functional response space than as a set of stable discrete phenotypes. Behavioural fingerprints derived from baseline dynamics and five perturbation scenarios formed a highly structured response space, with the first three principal components explaining 80.75% of the total variance. The response axes were associated with Gleason score and, more selectively, with TP53 mutation status, while individual digital twins differed in their responses to AR, PI3K, and TGF-β perturbations. The major variance structure was preserved across different strengths of transcriptome-informed parameter personalisation, whereas HDBSCAN cluster assignments were not. Additional sensitivity analyses showed that the dominant response structure was also preserved under uniform perturbation strengths and moderate global changes in model parameters. These findings support a continuous representation of functional heterogeneity in prostate cancer rather than its division into a small number of discrete response subtypes.

The organisation of the response space also helps explain how transcriptomic programmes are integrated by the model. PC1 accounted for 64.0% of behavioural variance and was strongly positively associated with most transcriptome-derived functional nodes, including AR, PI3K/AKT, MAPK/ERK, TGF-β, EMT/ECM, and TP53/apoptosis. Rather than reflecting a single signalling pathway, PC1 therefore represents a broad axis of coordinated functional activity. PC3 had a different profile, with a positive association with EMT/ECM and negative associations with cell-cycle/proliferation and DDR. These relationships are not independent biological validation because the transcriptomic node scores are upstream inputs to the digital twins. This dependence was confirmed by the direct static comparator: PC1 derived from the eight input node scores correlated very strongly with simulation-derived PC1 (ρ=0.976). Gleason score was also associated with static PC1 (ρ=0.337), slightly more strongly than with simulation-derived PC1 (ρ=0.300). The dominant response axis should therefore not be interpreted as new clinical information created by the dynamical model. Instead, the simulations re-express the molecular input within a common dynamical and counterfactual framework in which baseline behaviour and responses to defined perturbations can be compared across tumour samples. The secondary axes were more substantially reorganised: static PC2 correlated strongly with simulation-derived PC3 rather than PC2, indicating rotation of lower-variance structure after dynamical transformation. This interpretation is consistent with our previous analysis of prostate cancer signalling organisation, in which disease progression was associated with altered coordination between pathways despite comparatively modest changes in individual pathway activities [11]. The present analysis extends that concept from static pathway organisation to simulated functional responses.

The broad covariance among the transcriptome-derived nodes also raised the possibility that part of this structure reflected variation in tumour purity rather than tumour-intrinsic signalling alone. This was supported by negative correlations between CPE purity and several functional nodes, particularly EMT/ECM (ρ=−0.54), MAPK/ERK (ρ=−0.43), and PI3K/AKT (ρ=−0.42), as well as with simulation-derived PC1 (ρ=−0.44). Purity therefore contributes to the global molecular axis represented by PC1. It does not, however, provide a complete explanation for the observed structure. Adjustment for CPE reduced the median pairwise correlation among the eight nodes only modestly, from approximately 0.746 to 0.722, and the associations of Gleason score and TP53 status with the simulation-derived axes persisted after adjustment. ESTIMATE-derived purity produced the same overall interpretation. The global co-activation pattern should consequently be viewed as a mixture of coordinated transcriptomic programmes and sample-composition effects rather than being attributed exclusively to tumour-cell signalling.

The associations with clinical and genomic variables provide a separate context for interpreting these response axes. Gleason score was positively associated with PC1 and negatively associated with PC2 and PC3, indicating systematic variation in modelled functional behaviour with histopathological grade. The corresponding Kruskal–Wallis effect sizes were largest for PC1 ($\epsilon^{2}=0.084$) and smaller for PC2 ($\epsilon^{2}=0.031$) and PC3 ($\epsilon^{2}=0.033$). TP53 mutation status was associated with both PC1 and PC3 after correction for multiple testing. PTEN coding mutation status was not significantly associated with the first three principal components of the digital-twin behavioural fingerprints. However, this finding requires caution because only 16 of the 492 modelled tumours carried a retained PTEN mutation, and mutation status alone does not capture copy-number loss, an important mechanism of PTEN alteration in prostate cancer. The additional post hoc analysis identified a significant association between continuous PTEN copy-number values and PC1 (ρ=−0.238, FDR-adjusted $p=3.18\times 10^{-7}$), which was also observed across multiple deletion thresholds. Combined PTEN mutation and copy-number alteration status was associated with PC1–PC3. These findings suggest that copy-number information captures variation in the digital-twin behavioural space that is not adequately represented by PTEN mutation status alone. Nevertheless, the associations remain exploratory, and segment-based copy-number estimates do not establish functional PTEN inactivation or a causal relationship with the simulated responses. SPOP mutation status remained unassociated with the three principal simulation-derived axes. The relationship with TP53 is biologically plausible, as alterations in this gene have previously been linked to adverse molecular and clinical features in primary prostate cancer [12].

Importantly, neither Gleason score nor mutation status contributed to the construction of the behavioural fingerprints. These variables were also not used to derive the principal components. Their associations with the response space therefore emerged only after the digital-twin representations had been constructed. They should nevertheless be regarded as associations rather than external validation. They should also not be described as independent of the underlying transcriptomic state, given the close correspondence between static and simulation-derived PC1 and the contribution of tumour purity to both representations. In particular, only four tumours had a Gleason score of 10, and the present analysis does not establish that any response axis predicts treatment outcome, progression, or survival. The findings instead suggest that simulation-derived functional variation is related to established dimensions of prostate cancer biology and aggressiveness [13,14,15].

The perturbation experiments show how this functional variation translates into patient-specific responses. AR suppression primarily reduced AR activity, with a smaller decrease in proliferation. PI3K suppression had a different response profile, with its main effects seen in proliferation and survival. TGF-β induction was most clearly reflected in EMT. The largest median increase in EMT occurred when TGF-β induction was combined with AR suppression. These response patterns are consistent with the established role of AR signalling and its interaction with the PI3K/AKT pathway in PCa [16,17,18]. They also agree with the known involvement of TGF-β and EMT-related programmes in tumour plasticity and progression [19]. More relevant to the purpose of the digital-twin model, however, was the variation in response magnitude between tumour samples exposed to the same perturbation class. The proliferative response to AR suppression was inversely associated with the EMT response to TGF-β induction (ρ=−0.61). Tumour samples with a stronger proliferative response to AR perturbation therefore tended to show a weaker EMT response to TGF-β induction, and vice versa. This relationship suggests that responses to different perturbations were coordinated rather than varying independently.

Because perturbation strength in the primary model was itself dependent on the baseline state of the targeted node, this coupling could potentially have arisen from the intervention definition. The uniform-perturbation sensitivity analysis argues against this being the sole explanation. When identical AR, PI3K, and TGF-β perturbation factors were applied to every sample, perturbation-response features remained highly concordant with the primary model (median ρ=0.992), and the inverse proliferation–EMT relationship persisted (ρ=−0.679). PC1 and PC2 were also highly stable, although PC3 was sensitive to the perturbation formulation. Within the model, these patterns reflect differences in how sample-specific transcriptomic states shape the propagation of pathway perturbations. They remain simulation-derived responses, but they should not be interpreted as predictions of sensitivity to androgen-deprivation therapy, metastatic potential, or other clinical outcomes.

The direction of the principal AR-suppression responses nevertheless received partial experimental support from two independent LNCaP enzalutamide datasets. In both experiments, enzalutamide reduced androgen-response and E2F-target scores, consistent with the simulated reductions in AR activity and proliferation. Downstream correspondence was less complete: experimental EMT responses differed between datasets, DNA-repair scores decreased despite the near-zero simulated DDR response, and apoptosis changed in the opposite direction to the small modelled response. These comparisons therefore support selected response directions rather than validating the digital twin quantitatively. They also illustrate the expected gap between a compact tumour-level network model and cell-line experiments performed under different androgen stimuli and exposure durations.

The combined perturbations placed a further constraint on interpretation. The biological rationale for jointly perturbing AR and PI3K is supported by their reciprocal signalling interactions in prostate cancer and by experimental studies of combined AR and PI3K/AKT/mTOR targeting [18,20]. Departures from exact additivity were statistically consistent across tumour samples but small in magnitude. The largest relative deviation was 2.65%, for the proliferative response to combined AR and PI3K suppression, while all other interpretable deviations were approximately 1% or less. Because the simulations are deterministic, the small *p*-values primarily indicate consistency of the deviation across 492 simulated samples and should not be interpreted as estimates of model uncertainty. The magnitude of the deviation is therefore more informative than its statistical significance. We observed mostly additive effects of combined perturbations within the current network of modelled interactions. This near-additivity is also compatible with the parsimonious network design and the relatively limited cross-talk encoded between the perturbed nodes; it should not be regarded as an unexpected emergent property of prostate cancer biology. These results should not be confused with assessments of pharmacological synergy or antagonism, because the modelled quantifications of perturbations measure changes in activity of pathways, not effects of drugs. As such, the current model does not account for several important aspects of drug action, i.e., drug binding, exposure, dose–response relations, pharmacokinetics and pharmacodynamics. Extending the model to include drug-specific processes would allow formal evaluation of therapeutic interactions. However, such a model would also need to be calibrated and validated using appropriate experimental or clinical treatment-response data.

The sensitivity analysis showed a clear distinction between the continuous structure of the response space and its discretisation by clustering. Across personalisation strengths of λ=0.10–0.30, the first 15 principal components consistently retained more than 99% of behavioural-fingerprint variance. HDBSCAN, by contrast, identified two or three clusters at lower values of λ but no discrete clusters at λ=0.25 or 0.30, and agreement with the primary λ=0.20 assignment was limited outside the primary setting. We therefore do not regard the HDBSCAN solution as evidence for intrinsic prostate cancer response subtypes. More generally, an apparently distinct partition of high-dimensional biological data under one analytical parameterisation does not necessarily imply an underlying categorical biological structure. In the present analysis, the variance structure of the continuous response space was considerably more stable across personalisation strengths and therefore provides a more defensible description of model-derived functional heterogeneity.

Robustness to the numerical parameterisation of the shared network was evaluated separately. Global ±20% scaling of the regulatory weights or reference production parameters preserved the principal perturbation responses and the dominant response axis, with PC1 correlations of 0.995–0.999 relative to the primary model. The proliferation–EMT cross-perturbation relationship also retained its direction across all tested scenarios. Secondary PCs, particularly under changes in $\alpha_{0}$, were less stable. The primary conclusions therefore do not depend strongly on moderate global rescaling of the chosen parameters, but the lower-variance structure is more parameter dependent. This analysis is a local robustness assessment and does not resolve parameter identifiability or quantify the full uncertainty associated with the numerical model assumptions.

The personalisation ablation explains why transcriptomic information was incorporated into model parameters as well as initial conditions. When patient specificity was limited to the initial state ($x_{0}$), the baseline trajectories converged towards essentially the same steady state. Between-sample standard deviations across the principal functional outputs were approximately $10^{-8}$, indicating that little of the initial heterogeneity persisted at equilibrium. This behaviour is consistent with the structure of the deterministic model. With shared parameters, different initial conditions can follow different trajectories but ultimately converge towards the same attractor. The convergence observed in the ablation is therefore an expected consequence of the shared dynamical parameterisation rather than an independent biological finding. Continuous transcriptome-informed modulation of basal activity parameters ($\alpha_{i}$) preserved substantial inter-sample differences at steady state and reorganised the behavioural response space without altering the shared network topology or decay parameters. The differences in the initial states $x_{0}$ of a patient-specific model as introduced in this work were not sufficient to maintain differences between individual tumour samples for an extended period of time. However, inter-sample differences were maintained when basal activity parameters were additionally personalised using transcriptomic data. A patient-specific model is not only defined by the initial state of the system but also by how the system behaves under given input [21,22,23]. Hence, patient specificity is encoded not only in the initial molecular state represented by a tumour sample but also in the system dynamics.

Pathway-level abstraction made patient-specific dynamical modelling computationally tractable while retaining biological interpretability. Rather than modelling thousands of genes directly, transcriptomic measurements were first condensed into Reactome pathway activities and then mapped to eight functional network nodes. This reduction necessarily removes molecular detail, but it provides a defined state space in which perturbations and their downstream consequences can be simulated explicitly [24,25]. The pathway-mapping sensitivity analyses indicated that the node definitions were generally robust to the examined modifications. Excluding the 12 Reactome pathways shared between nodes resulted in minimal changes to the original node scores (Spearman ρ=0.985–1.000), while the AR node remained relatively stable in the leave-one-pathway-out analysis (median ρ=0.924). Comparison with the independently defined MSigDB Hallmark Androgen Response signature showed moderate concordance (ρ=0.421), suggesting that the Reactome-derived AR node captures aspects of androgen-related transcriptional activity but is not interchangeable with an established AR-response signature. These findings support the stability of the selected pathway mapping under the examined conditions, while highlighting the dependence of node definitions on pathway annotation and composition.

The resulting 42-feature behavioural fingerprint describes how each digital twin responded to the same set of perturbations. Applying a common perturbation panel across the cohort provided a common basis for comparing patient-specific trajectories and terminal states. The static comparator is important in defining what this response representation adds. It does not replace the underlying molecular profile or consistently improve its association with clinical variables. Instead, it places the molecular state within an explicit dynamical system and generates counterfactual response features that cannot be obtained from static PCA alone. In this sense, response-based characterisation adds a functional dimension to molecular profiling rather than replacing it.

Several limitations should be considered. The digital twins were derived from bulk tumour transcriptomics and therefore do not explicitly capture cellular composition, spatial organisation, stromal interactions, or intratumour subclonality. The purity analysis confirms that cellular composition contributes measurably to the transcriptomic node scores and to the principal response axes. Although the main Gleason and TP53 associations persisted after purity adjustment, bulk-derived model states cannot be interpreted as tumour-cell-intrinsic measurements. The eight-node network is also deliberately compact, with a predefined set of regulatory interactions. Simulated responses are therefore conditional on this topology and do not represent signalling mechanisms outside the modelled network.

A further source of abstraction is the predefined mapping of Reactome pathways to the eight functional nodes. Pathways can have overlapping or context-dependent biological roles, and alternative pathway-to-node assignments could therefore produce different model states and simulated responses. The overlap-exclusion, AR leave-one-pathway-out, and Hallmark AR analyses reduce this concern for the principal node definitions but do not exhaust the possible biologically reasonable mappings. Importantly, these sensitivity analyses assessed the stability of transcriptome-derived node scores rather than the downstream simulated responses; therefore, the robustness of the complete behavioural response space to alternative pathway-to-node mappings remains to be established. The current simulations are also deterministic and do not represent stochastic cellular behaviour.

The model does not include pharmacokinetics, pharmacodynamics, drug-specific mechanisms, or dose–exposure relationships. Its patient-specific parameterisation also relies on modelling assumptions. Transcriptomic data were used both to initialise the system and to personalise basal activity parameters. Although the ablation analysis supports the need for parameter-level personalisation to preserve inter-sample differences at steady state, the exponential mapping between standardised node scores and $\alpha_{i}$ has not been experimentally calibrated. Likewise, the numerical interaction weights, reference production parameters, decay rate, and Hill-function parameters were selected as modelling assumptions rather than estimated from TCGA or kinetic experiments. The ±20% sensitivity analysis supports the stability of the dominant response structure around these choices but does not constitute global uncertainty analysis or parameter calibration. Similarly, the continuous variance structure was retained across the tested range of personalisation strengths, whereas discrete cluster solutions remained parameter dependent.

The clinical analyses were largely restricted to Gleason score and selected recurrent mutations. The additional PTEN copy-number analysis broadened the genomic assessment and identified associations not evident from mutation status alone. However, these findings were derived from a post hoc analysis of the same TCGA cohort and were not independently validated. Moreover, segment-based copy-number estimates do not directly establish PTEN protein loss or functional inactivation. Treatment response, biochemical recurrence, disease progression, survival, and longitudinal molecular measurements were not available for evaluation within the present framework. The study was also based on a single patient-derived transcriptomic cohort. The two independent enzalutamide datasets provide experimental support for the direction of selected AR-suppression responses, but they are LNCaP cell-line experiments and cannot establish reproducibility of the sample-level response landscape in an independent patient cohort. Independent molecular cohorts, longitudinal sampling, and experimentally observed perturbation responses will therefore be required to determine whether the simulated response axes are reproducible across datasets and whether they have predictive clinical value.

The present results support perturbation-informed digital twins as a means of representing prostate cancer heterogeneity in functional, rather than exclusively static molecular, terms. The main contribution is not a new classification into discrete prostate cancer subtypes, but a patient-specific response space in which molecular differences are translated into quantitatively comparable simulated behaviours. The close correspondence between static and simulation-derived PC1 further defines the scope of this contribution: the framework should be viewed as a mechanistic re-expression of transcriptomic heterogeneity that enables explicit counterfactual perturbation analysis, rather than as a method that generates an independent dominant molecular or clinical signal.

Moving from this functional representation towards treatment-specific prediction would require additional evidence. Longitudinal patient data and independent cohorts would be needed to test whether the simulated response patterns are reproducible and clinically informative. Treatment modelling would also require experimentally calibrated perturbations and explicit representation of drug-specific pharmacokinetics and pharmacodynamics. More broadly, clinical translation would depend on model calibration, independent validation, and uncertainty quantification, consistent with current recommendations for patient-specific mechanistic models and medical digital twins [26,27].

Because the models share the same regulatory parameters, patient-specific initial conditions primarily influence transient dynamics rather than long-term equilibrium states. Similarly, the near-additive responses to combined perturbations are consistent with the relatively weak AR–PI3K/AKT interactions specified in the model and should not be interpreted as independent evidence of biological additivity.

At its present stage, the framework should therefore be viewed as a mechanistic tool for exploring patient-specific functional heterogeneity and generating testable hypotheses, rather than as a validated model for predicting treatment response or supporting clinical decisions.

## 4. Materials and Methods

### 4.1. Study Design and Computational Workflow

The study was designed as a retrospective computational analysis of prostate cancer transcriptomic data. The workflow comprised six main stages: (i) preprocessing of tumour transcriptomic data; (ii) calculation of Reactome pathway activity scores and their aggregation into functional model nodes; (iii) construction of sample-specific dynamical models; (iv) simulation of baseline and perturbed states; (v) construction of behavioural fingerprints and analysis of the resulting functional response space; and (vi) post-hoc clinical and molecular association analyses. Additional analyses were performed to examine specific assumptions and sources of uncertainty in the framework, including comparison with the underlying static transcriptomic representation, tumour-purity adjustment, uniform-perturbation and model-parameter sensitivity analyses, pathway-mapping robustness, combined-perturbation non-additivity, personalisation ablation, PTEN copy-number analysis, and comparison with independent experimental AR-suppression datasets.

The final analytical cohort comprised 492 primary TCGA-PRAD tumour samples from 488 unique TCGA cases. Four cases contributed two primary-tumour samples. Clinical variables and mutation status were not used to construct the digital twins or behavioural fingerprints and were introduced only in post-hoc association analyses. Copy-number and tumour-purity annotations were likewise used only in post-hoc analyses and did not contribute to model construction or parameterisation.

### 4.2. Data Acquisition and Cohort Harmonisation

Transcriptomic and somatic mutation data were obtained from the Genomic Data Commons (GDC) for The Cancer Genome Atlas Prostate Adenocarcinoma project (TCGA-PRAD) on 1 February 2026, as established from the local creation and modification timestamps of the downloaded files. The original GDC file identifiers and associated processing workflow metadata were retained to support data provenance and reproducibility. Open-access RNA-sequencing files were queried through the GDC application programming interface using the data type Gene Expression Quantification, experimental strategy RNA-Seq, and workflow type STAR-Counts. The RNA-sequencing data comprised 554 STAR Counts files annotated using GENCODE v36. For each downloaded STAR–Counts file, the unstranded count column was indexed by gene_name. Duplicate gene names within individual files were collapsed by summing their counts, and individual files were combined into a gene-by-sample expression matrix. Rows without an annotated gene symbol were excluded before aggregation. The resulting matrix contained 59,427 unique gene symbols.

The processing pipeline included a zero-filling operation following the combination of individual expression profiles. To verify its effect, all 554 original STAR Counts files were retrospectively audited. All files contained the same set of 59,427 gene symbols, and no missing count values were identified. Consequently, the zero-filling operation did not replace any values or modify the resulting expression matrix. No missing patient-level clinical measurements or unavailable molecular samples were imputed.

Expression files were mapped to TCGA sample identifiers using the case and sample metadata returned by the GDC. Only samples annotated as Primary Tumor were retained. The resulting expression dataset comprised 501 unique primary tumour samples. No duplicate sample identifiers were identified among these samples, and therefore no aggregation of counts across multiple expression files was required for the primary tumour cohort.

Open-access masked somatic mutation files were obtained from the same TCGA-PRAD project in Mutation Annotation Format (MAF). Sample barcodes were standardised to the 16-character TCGA sample level. The mutation analysis retained Frame Shift Deletion, Frame Shift Insertion, In-Frame Deletion, In-Frame Insertion, Missense Mutation, Nonsense Mutation, Nonstop Mutation, Splice Site, Translation Start Site, and Splice Region events. Where the corresponding MAF fields were available, variants were additionally filtered to retain records passing the supplied quality filter, records annotated as somatic or without an available mutation-status annotation, and variants with a gnomAD allele frequency below 0.001 or without an available gnomAD frequency.

For each sample and gene, a binary mutation indicator was defined as 1 when at least one retained mutation event was present and 0 otherwise. The analytical cohort was defined as the intersection of primary-tumour samples represented in the RNA-sequencing expression matrix and samples represented in the filtered binary mutation matrix. Of the 501 unique primary tumour samples identified in the RNA-seq dataset, 499 had corresponding somatic mutation data available in the GDC MAF metadata. Two samples were excluded because no corresponding MAF files were available. Among the remaining 499 samples, five had MAF files containing no reported variant records, while two contained only synonymous (silent) variants, which were excluded by the variant-filtering criteria. These seven samples were therefore not represented in the mutation matrix used for downstream analyses. Following harmonisation of gene expression and somatic mutation data, the final analytical cohort comprised 492 primary tumour samples from 488 unique patients. Four patients contributed two primary tumour samples each (01A and 01B). Both samples were retained where the corresponding molecular data were available, and the analyses were therefore conducted at the tumour-sample level.

PTEN copy-number status was evaluated post hoc and was not used for digital-twin construction. Copy-number segment data generated using the DNAcopy pipeline on GRCh38 were obtained from GDC for primary TCGA-PRAD tumours. For each sample, PTEN copy number was summarised across the GRCh38 PTEN locus (chromosome 10: 87,863,625–87,971,930) as the overlap-length-weighted mean segment value. Copy-number estimates could be matched to 489 of the 492 modelled tumour samples. The continuous PTEN copy-number value was used as the primary copy-number measure, with threshold-based deletion definitions examined as sensitivity analyses.

### 4.3. Transcriptomic Preprocessing

The harmonised gene-count matrix comprised 59,427 genes across 492 tumour samples. Raw counts were normalised to counts per million (CPM) separately for each sample. For gene *g* in sample *p*, CPM was calculated as

(1) $${\mathrm{CPM}}_{g,p}=\frac{C_{g,p}}{\sum_{h=1}^{G}C_{h,p}}\times 10^{6},$$

where $C_{g,p}$ denotes the unstranded STAR count for gene *g* in sample *p*, and the denominator represents the total library size of that sample.

Low-expression genes were removed before pathway-level analysis. A gene was retained if it reached a CPM value of at least 1 in at least 10 tumour samples:

(2) $$\sum_{p=1}^{N}1\left({\mathrm{CPM}}_{g,p}\geq 1\right)\geq 10,$$

where 1(·) denotes the indicator function and N=492. This filtering retained 20,647 genes.

The retained CPM values were subsequently transformed using a pseudocount of 1:

(3) $$E_{g,p}=\log_{2}\left({\mathrm{CPM}}_{g,p}+1\right).$$

The resulting log_2_(CPM + 1) expression matrix was used as the transcriptomic input for downstream Reactome pathway activity scoring.

### 4.4. Reactome Pathway Activity Scoring

Pathway-level activity scores were derived from the filtered log_2_(CPM + 1) expression matrix using Reactome gene sets obtained in Gene Matrix Transposed (GMT) format. Before pathway scoring, expression values for each gene were standardised across the 492 tumour samples:

(4) $$Z_{g,p}=\frac{E_{g,p}-{\bar{E}}_{g}}{s_{g}},$$

where $E_{g,p}$ is the log_2_(CPM + 1) expression of gene *g* in sample *p*, ${\bar{E}}_{g}$ is its mean expression across the cohort, and $s_{g}$ is the corresponding sample standard deviation. Genes with zero variance across the cohort were assigned a standardised value of zero.

For each Reactome pathway, duplicate gene symbols within the corresponding GMT gene set were removed while preserving their original order, and the remaining genes were matched to the expression matrix. Pathways with between 10 and 300 matched genes, inclusive, were retained. Of 2830 Reactome gene sets evaluated, 1628 satisfied this criterion and were included in the subsequent analysis.

The activity score of pathway *r* in sample *p* was calculated as the arithmetic mean of the standardised expression values of its matched member genes:

(5) $$P_{r,p}=\frac{1}{|G_{r}|}\sum_{g\in G_{r}}Z_{g,p},$$

where $G_{r}$ denotes the set of genes from pathway *r* represented in the filtered expression matrix. The resulting matrix contained activity scores for 1628 Reactome pathways across 492 tumour samples. These 1628 pathways constituted the scored pathway pool from which pathways satisfying the predefined functional-node mapping criteria were subsequently selected; the eight digital-twin nodes were therefore constructed from a subset of these pathways rather than from all 1628 scored pathways.

### 4.5. Aggregation of Pathway Activities into Functional Model Nodes

Reactome pathway activities were aggregated into eight functional nodes representing signalling and cellular programmes relevant to prostate cancer: AR programme, PI3K/AKT, MAPK/ERK, TGF-β, EMT/ECM/invasion, cell-cycle/proliferation, apoptosis/TP53, and DNA-damage response (DDR). Pathways were assigned to nodes using predefined, case-insensitive regular-expression rules applied to Reactome pathway names. The rules were designed to identify pathways whose names corresponded to the biological processes represented by each node, providing a reproducible, biologically informed mapping rather than an outcome-driven selection of pathways.

For each node *i* and patient *p*, the node score was calculated as the median activity across all Reactome pathways assigned to that node:

(6) $$S_{i,p}={\mathrm{median}}_{r\in R_{i}}\left(P_{r,p}\right),$$

where $R_{i}$ denotes the set of Reactome pathways assigned to node *i* and $P_{r,p}$ is the pathway activity score defined above. Median aggregation was used to reduce the influence of individual pathway-level extremes.

The final mapping comprised 232 pathway-to-node assignments representing 220 unique Reactome pathways (see Supplementary Materials, Table S1). The numbers of pathways assigned to the individual nodes were 5 for the AR programme, 22 for PI3K/AKT, 42 for MAPK/ERK, 9 for TGF-β, 33 for EMT/ECM/invasion, 50 for cell-cycle/proliferation, 38 for apoptosis/TP53, and 33 for DDR. Twelve Reactome pathways were assigned to two functional nodes because their names matched the predefined criteria for both biological programmes; no pathway was assigned to more than two nodes. The resulting eight node scores for each of the 492 tumours constituted the transcriptome-derived molecular representation used for digital-twin parameterisation. The distributions of the resulting transcriptome-derived functional-node scores are summarised in Appendix A, Table A1.

To assess sensitivity to pathway overlap and to the relatively small number of pathways contributing to the AR-programme node, three mapping-robustness analyses were performed. First, node scores were recalculated after excluding the 12 Reactome pathways assigned to more than one functional node, and the resulting scores were compared with the primary node definitions using Spearman rank correlations. Second, the AR-programme node was recalculated in a leave-one-pathway-out analysis in which each of its five constituent Reactome pathways was removed in turn, with the resulting scores compared against the original AR-programme score. Third, the Reactome-derived AR-programme score was compared with an alternative androgen-response representation based on the independently defined MSigDB HALLMARK_ANDROGEN_RESPONSE gene set. The same gene-wise standardisation and mean gene-set scoring procedure used for the primary pathway analysis was applied. Of the 101 genes in the Hallmark set, 100 were represented in the filtered expression matrix. The resulting Hallmark score was compared with each of the eight Reactome-derived functional-node scores using Spearman rank correlations. Because this benchmark was derived from the same TCGA-PRAD expression cohort, it was treated as a robustness analysis using an independently defined gene set rather than as external validation.

### 4.6. Patient-Specific Digital-Twin Parameterisation

The eight transcriptome-derived functional-node scores were used to personalise each digital twin through two complementary mechanisms: patient-specific initial states and patient-specific basal production parameters. The raw node scores were used to define the initial state of each model node, whereas cohort-standardised node scores were used to modulate its basal production strength. Network topology and decay parameters were shared across all patients.

For tumour sample *p*, the raw score $S_{i,p}$ of node *i* was transformed to the bounded initial state

(7) $$x_{i,p}\left(0\right)=\frac{1}{1+\exp(-S_{i,p})}.$$

Thus, transcriptome-derived node scores were mapped onto the interval (0,1) without imposing discrete molecular classes.

For parameter personalisation, each node score was independently standardised across the 492-sample cohort:

(8) $$Z_{i,p}=\frac{S_{i,p}-{\bar{S}}_{i}}{s_{i}},$$

where ${\bar{S}}_{i}$ and $s_{i}$ denote the cohort mean and sample standard deviation, respectively, for node *i*. The standardised scores were used only for parameter personalisation; the unstandardised scores were retained for construction of the initial states.

The patient-specific basal production parameter was defined as

(9) $$\alpha_{i,p}=\alpha_{0,i}\exp\left[\lambda_{\alpha }\mathrm{clip}\left(Z_{i,p},-z_{\mathrm{clip}},z_{\mathrm{clip}}\right)\right],$$

where $\alpha_{0,i}$ is the shared reference production parameter for node *i*, $\lambda_{\alpha }=0.20$ controls the strength of transcriptome-informed personalisation, and $z_{\mathrm{clip}}=2.5$ limits the influence of extreme standardised node scores. This exponential mapping preserves positive production parameters while providing a continuous monotonic relationship between transcriptomic activity and node-specific production strength.

The reference production parameters $\alpha_{0,i}$ were 0.60 for the AR programme, 0.50 for PI3K/AKT, 0.35 for MAPK/ERK, 0.35 for TGF-β, 0.30 for EMT/ECM/invasion, 0.45 for cell-cycle/proliferation, 0.35 for apoptosis/TP53, and 0.35 for DDR. Somatic mutation status was not used to modify initial states, model parameters, network interactions, or perturbation strengths in the primary digital-twin model. PTEN, TP53, and SPOP mutation indicators were retained exclusively for subsequent post-hoc molecular association analyses. PTEN copy-number status was likewise not used for model construction or parameterisation.

### 4.7. Dynamical Model

Each sample-specific digital twin was represented as an eight-node dynamical system comprising the AR programme, PI3K/AKT, MAPK/ERK, TGF-β, EMT/ECM/invasion, cell-cycle/proliferation, apoptosis/TP53, and DDR nodes. The dynamics of node *i* in tumour sample *p* were defined as

(10) $$\frac{dx_{i,p}}{dt}=\alpha_{i,p}e_{i,p}\sigma \left(\sum_{j=1}^{8}W_{ij}H\left(x_{j,p}\right)\right)\left(1-x_{i,p}\right)-\delta_{i}x_{i,p}.$$

Here, $x_{i,p}$ is the state of node *i* for tumour sample *p*, $\alpha_{i,p}$ is the transcriptome-informed basal production parameter defined above, $e_{i,p}$ is a multiplicative perturbation factor, $W_{ij}$ is the directed interaction weight from node *j* to node *i*, and $\delta_{i}$ is the node-specific decay parameter. Under baseline conditions, all perturbation factors were set to $e_{i,p}=1$.

Regulatory input was transformed using the logistic function

(11) $$\sigma \left(u\right)\frac{1}{1+\exp(-u)},$$

whereas the activity of each upstream node entered the regulatory term through a Hill function:

(12) $$H\left(x\right)\frac{x^{n}}{k^{n}+x^{n}},$$

with Hill coefficient n=2 and half-activation constant k=0.5.

The reference production parameters, decay parameters, and non-zero directed regulatory interactions defining the shared dynamical model are summarised in Table 1. All nodes used a common decay parameter of $\delta_{i}=0.60$. Interaction weights were positive for activating relationships and negative for inhibitory relationships; all interactions not specified in Table 1 were set to zero. The network topology, reference production parameters, interaction weights, and decay parameters were identical across patients. Patient specificity entered the dynamical system through the transcriptome-derived initial states and basal production parameters described in Section 4.6. The qualitative network topology and signs of the regulatory relationships were selected to represent established interactions among the modelled prostate-cancer signalling programmes. The numerical interaction weights, reference production parameters, common decay rate, and Hill-function parameters were modelling assumptions used to define a parsimonious shared dynamical system; they were not fitted to the TCGA cohort and should not be interpreted as experimentally estimated kinetic constants. Their influence on the principal results was therefore examined in a dedicated parameter-sensitivity analysis described below.

For each baseline or perturbed condition, the system was numerically integrated from t=0 to t=30 using the adaptive Runge–Kutta method implemented by scipy.integrate.solve_ivp with the RK45 solver. Trajectories were evaluated at 301 equally spaced time points using relative and absolute tolerances of $10^{-6}$ and $10^{-8}$, respectively. During evaluation of the ODE right-hand side, the state vector supplied to the regulatory and production terms was clipped to [0,1] as a numerical safeguard. No post-integration clipping was applied to the solver-returned trajectories. A complete audit of all 2952 primary simulations (492 samples × six conditions; 7,108,416 returned state values) identified no state outside [0,1], either strictly or using a numerical tolerance of $10^{-12}$. The observed state range was 0.0882–0.8376. Thus, the solver-returned trajectories remained within the intended state domain without requiring post hoc truncation.

### 4.8. In Silico Perturbation Panel and Functional Outputs

Each sample-specific digital twin was simulated under baseline conditions and five predefined perturbation scenarios: AR suppression, PI3K/AKT suppression, TGF-β induction, combined AR and PI3K/AKT suppression, and combined AR suppression with TGF-β induction. Perturbations were implemented through the multiplicative factor $e_{i,p}$ in the node production term. All nodes not targeted by a given perturbation retained $e_{i,p}=1$.

Perturbation strengths were continuously adapted to the patient-specific initial state of the corresponding target node. For AR suppression, the AR-programme perturbation factor was defined as

(13) $$e_{\mathrm{AR},p}=0.55-0.40x_{\mathrm{AR},p}\left(0\right),$$

yielding a factor between 0.15 and 0.55.

PI3K/AKT suppression was defined as

(14) *e*_PI3K,*p*_ = 0.70 − 0.35*x*_PI3K,*p*_(0),

yielding a factor between 0.35 and 0.70.

TGF-β induction was defined as

(15) *e*_TGF*β*,*p*_ = 1.20 + 0.80[1 − *x*_TGF*β*,*p*_(0),

yielding a factor between 1.20 and 2.00.

The two combination conditions applied the corresponding patient-specific single-node perturbation factors simultaneously. No additional interaction term was introduced for combined perturbations. The complete perturbation panel, including the baseline condition and the two combined perturbation scenarios, is summarised in Table 2. The state-dependent perturbation formulation was intended to represent a relative intervention whose effective strength depends continuously on the baseline state of the targeted programme rather than an identical intervention magnitude across all tumours. Because this design could itself contribute to between-sample differences in simulated response, a uniform-perturbation sensitivity analysis was additionally performed. In this analysis, every tumour received the cohort-median perturbation factor from the primary model for each target: 0.3451 for AR suppression, 0.5199 for PI3K/AKT suppression, and 1.5839 for TGF-β induction. Combined perturbations used the corresponding uniform factors simultaneously. Initial states, model parameters, simulation settings, fingerprint construction, and downstream analyses were otherwise unchanged.

Five functional outputs were extracted from the terminal state of each simulation. AR activity, proliferative activity, EMT activity, and DDR activity were defined as the terminal states of the corresponding model nodes:

(16) $$A_{\mathrm{AR},p}=x_{\mathrm{AR},p}\left(t_{\mathrm{end}}\right),$$

(17) $$P_{\mathrm{prolif},p}=x_{\mathrm{prolif},p}\left(t_{\mathrm{end}}\right),$$

(18) $$E_{\mathrm{EMT},p}=x_{\mathrm{EMT},p}\left(t_{\mathrm{end}}\right),$$

and

(19) $$D_{\mathrm{DDR},p}=x_{\mathrm{DDR},p}\left(t_{\mathrm{end}}\right).$$

Survival was represented as the complement of the terminal apoptosis/TP53-node state:

(20) *S*_survival,*p*_ = 1 − *x*_Apoptosis/TP53,*p*_(*t*_end_).

In addition to the five terminal-state outputs, two trajectory-level descriptors were calculated for each simulation. Settling time was defined as the earliest sampled time point $t_{k}$ at which the total absolute deviation of the eight-dimensional state vector from its terminal state,

(21) $$D\left(t_{k}\right)=\sum_{i=1}^{8}\left|x_{i}\left(t_{k}\right)-x_{i}\left(t_{\mathrm{end}}\right)\right|,$$

was less than or equal to $10^{-3}$ for at least 20 consecutive sampled time points. If this condition was not satisfied during the simulation, the settling time was assigned the terminal simulation time, $t_{\mathrm{end}}=30$.

The mean absolute derivative was calculated from successive sampled states as

(22) $$M=\frac{1}{K-1}\sum_{k=1}^{K-1}\sum_{i=1}^{8}\frac{\left|x_{i}\left(t_{k+1}\right)-x_{i}\left(t_{k}\right)\right|}{t_{k+1}-t_{k}},$$

where K=301 is the number of sampled time points. This descriptor therefore summarised the overall magnitude of state change across all eight model nodes over the simulated trajectory. Together, the five terminal-state outputs and two trajectory-level descriptors provided seven functional measures for each simulated condition.

### 4.9. Behavioural Fingerprint Construction

For each sample-specific digital twin, simulation results from the baseline condition and the five perturbation scenarios were combined into a behavioural fingerprint. Seven measures were extracted from each simulation: AR activity, proliferative activity, EMT activity, survival, DDR activity, settling time, and mean absolute derivative.

The seven baseline measures were retained directly as components of the fingerprint. For each of the five perturbation scenarios, the response of measure *m* was expressed as the within-sample change from the corresponding baseline value:

(23) $$\Delta F_{m,p}^{\left(q\right)}=F_{m,p}^{\left(q\right)}-F_{m,p}^{\left(\mathrm{baseline}\right)},$$

where $F_{m,p}^{\left(q\right)}$ denotes functional measure *m* for tumour sample *p* under perturbation *q*, and $F_{m,p}^{\left(\mathrm{baseline}\right)}$ denotes the corresponding baseline measure for the same sample.

The complete behavioural fingerprint therefore comprised seven baseline features and seven response features for each of five perturbation conditions:

(24) 7+(5×7)=42

features per tumour sample. The perturbation conditions were AR suppression, PI3K/AKT suppression, TGF-β induction, combined AR and PI3K/AKT suppression, and combined AR suppression with TGF-β induction.

Somatic mutation indicators for PTEN, TP53, and SPOP were retained as sample-level annotations but were not included among the 42 behavioural features.

### 4.10. Principal-Component Analysis of the Functional Response Space

The 42-dimensional behavioural fingerprints were used to construct a low-dimensional representation of inter-sample variation in simulated functional responses. Before dimensionality reduction, each behavioural feature was independently centred and scaled across the 492 tumour samples using StandardScaler from scikit-learn, which uses the cohort mean and population standard deviation (equivalent to ddof=0):

(25) $${\tilde{F}}_{k,p}=\frac{F_{k,p}-\bar{F}k}{\sigma F_{k}},$$

where $F_{k,p}$ is behavioural feature *k* for tumour sample *p*, and $\bar{F}k$ and $\sigma F_{k}$ are the corresponding cohort mean and population standard deviation, respectively.

Principal-component analysis (PCA) was subsequently performed on the standardised 492×42 behavioural-feature matrix using the implementation in scikit-learn. Fifteen principal components were retained for quantitative characterisation of the response-space variance structure. Sample scores on the first three principal components (PC1–PC3) were used as the principal low-dimensional representation of the functional response landscape. Together, these three components accounted for 80.75% of the total variance in the standardised behavioural fingerprints.

To aid biological interpretation of the resulting response axes, PC1–PC3 scores were compared with the eight transcriptome-derived functional-node scores used to personalise the digital twins. For each principal component *c* and functional node *i*, the association was quantified using Spearman’s rank correlation coefficient:

(26) $$\rho_{c,i}=\mathrm{Spearman}\left({\mathrm{PC}}_{c},S_{i}\right).$$

These correlations were used as descriptive measures linking variation in simulated response behaviour to the transcriptomic inputs of the model. Because the functional-node scores contributed directly to digital-twin personalisation, these analyses were interpreted as characterisation of how the transcriptomic input was re-expressed in the simulated response space rather than as independent molecular validation.

### 4.11. Static Transcriptomic Comparator Analysis

To determine how much of the simulation-derived response-space structure was already present in the transcriptomic inputs, an analogous PCA was performed directly on the eight functional-node scores before dynamical simulation. The eight node scores were standardised across the 492 tumour samples and subjected to PCA using the same scikit-learn implementation. Scores on the first three static principal components were retained. Associations of static PC1–PC3 with Gleason score and PTEN, TP53, and SPOP mutation status were evaluated using the same statistical procedures applied to the simulation-derived PCs. Spearman rank correlations were used for Gleason score, and two-sided Mann–Whitney *U* tests with rank-biserial effect sizes were used for mutation status. Correspondence between the static and simulation-derived principal components was additionally assessed using Spearman rank correlations. This analysis was used to distinguish structure already present in the static transcriptomic-node representation from the functional re-expression generated by the dynamical simulation layer.

### 4.12. Clinical and Molecular Association Analyses

Associations between the principal functional-response axes and clinicomolecular characteristics were evaluated post hoc. Analyses were restricted to PC1–PC3, which together captured the dominant structure of the simulated behavioural response space.

Gleason scores were obtained at the sample level and defined as the sum of the primary and secondary Gleason patterns. The monotonic association between each principal-component score and Gleason score was assessed using Spearman’s rank correlation:

(27) $$\rho_{c}=\mathrm{Spearman}\left({\mathrm{PC}}_{c},G\right),$$

where ${\mathrm{PC}}_{c}$ denotes the tumour-sample score on principal component *c* and *G* denotes the Gleason score. In addition, differences in PC scores among the observed Gleason-score groups were evaluated using the Kruskal–Wallis test. Descriptive statistics, including sample count, median, mean, and standard deviation of PC1–PC3, were calculated separately for each Gleason-score group.

For each Kruskal–Wallis comparison, effect magnitude was additionally quantified using epsilon-squared,

(28) $$\epsilon^{2}=\frac{H-k+1}{n-k},$$

where *H* is the Kruskal–Wallis statistic, *k* is the number of Gleason-score groups, and *n* is the number of observations included in the comparison.

Molecular associations were evaluated for PTEN, TP53, and SPOP somatic mutation status. For each gene and each of PC1–PC3, PC-score distributions in mutation-positive and mutation-negative tumours were compared using a two-sided Mann–Whitney *U* test. Effect size was quantified using the rank-biserial correlation:

(29) $$r_{\mathrm{rb}}=\frac{2U}{n_{\mathrm{mut}}n_{\mathrm{wt}}}-1,$$

where *U* is the Mann–Whitney statistic calculated with the mutation-positive group as the first group, and $n_{\mathrm{mut}}$ and $n_{\mathrm{wt}}$ denote the numbers of mutation-positive and mutation-negative tumours, respectively. Positive values therefore indicate higher PC scores in the mutation-positive group, whereas negative values indicate lower scores.

Multiple-testing correction was performed using the Benjamini–Hochberg false-discovery-rate (FDR) procedure. Correction was applied separately across the three PC–Gleason Spearman tests and the three Kruskal–Wallis tests, and jointly across the nine mutation-association tests corresponding to three genes and three principal components. Both nominal and FDR-adjusted *p*-values were retained.

### 4.13. Tumour-Purity Sensitivity Analysis

Potential confounding by tumour purity was examined using published TCGA tumour-purity estimates from Aran et al. [28]. Consensus Purity Estimate (CPE) values were used as the primary purity measure because ABSOLUTE estimates were not available for TCGA-PRAD in the retrieved reference dataset. CPE values could be matched to 487 of the 492 tumour samples. ESTIMATE-derived purity values, available for 488 samples, were used as an additional sensitivity measure. Spearman rank correlations were calculated between tumour purity and each of the eight transcriptome-derived functional-node scores and between tumour purity and PC1–PC3 from both the static and simulation-derived representations. To determine whether the observed Gleason associations persisted after accounting for purity, partial Spearman correlations were calculated between Gleason score and each PC while controlling for the corresponding purity estimate. Mutation associations were additionally evaluated in regression models including tumour purity as a covariate. These analyses were restricted to samples for which the corresponding purity annotation was available and were treated as sensitivity analyses rather than as part of digital-twin construction.

### 4.14. PTEN Copy-Number Analysis

Because PTEN alterations in prostate cancer are not fully captured by coding mutation status, an additional post hoc analysis was performed using GDC copy-number segment data generated with the DNAcopy pipeline (GRCh38). For each tumour sample, PTEN copy number was summarised across the PTEN locus (chromosome 10: 87,863,625–87,971,930) as the overlap-length-weighted mean of the segment-level Segment_Mean values. Copy-number estimates were available for 489 of the 492 modelled tumour samples.

Associations with the first three principal components (PC1–PC3) were examined using the same 42-feature digital-twin behavioural fingerprints as in the primary analysis, encompassing baseline model characteristics and simulated perturbation responses. The features were standardised before principal component analysis, and mutation annotations were excluded from PCA construction. Spearman’s rank correlation was used to assess associations between continuous PTEN copy-number values and PC1–PC3.

As a sensitivity analysis, PTEN copy-number loss was defined using thresholds of ≤−0.2, ≤−0.3, ≤−0.4, and ≤−0.5 applied to the weighted segment values. Principal-component scores were compared between deletion-positive and deletion-negative samples using two-sided Mann–Whitney *U* tests, with rank-biserial correlations reported as effect sizes. Combined PTEN alteration status was defined as the presence of a retained somatic PTEN mutation or copy-number loss at the corresponding threshold and was evaluated using the same statistical approach. Multiple-testing correction was performed using the Benjamini–Hochberg false discovery rate procedure. Copy-number annotations were used exclusively for post hoc molecular interpretation and did not contribute to digital-twin construction or parameterisation.

### 4.15. Personalisation-Strength Sensitivity Analysis

The robustness of the functional response structure to the strength of transcriptome-informed parameter personalisation was evaluated by varying $\lambda_{\alpha }$, which controls the influence of the standardised functional-node scores on the sample-specific production parameters. The complete fingerprint-generation and downstream clustering pipeline was independently repeated for

(30) $$\lambda_{\alpha }\in \left\{0.10,\;0.15,\;0.20,\;0.25,\;0.30\right\},$$

with $\lambda_{\alpha }=0.20$ corresponding to the primary model.

For each value of $\lambda_{\alpha }$, the 42 behavioural features were independently standardised across the cohort and subjected to principal-component analysis. Fifteen principal components were retained, and their cumulative explained variance was recorded to assess preservation of the dominant continuous variance structure across personalisation strengths.

As a secondary assessment of whether the response space supported stable discrete partitions, HDBSCAN clustering was performed on the 15-dimensional PCA representation. HDBSCAN was configured with a minimum cluster size of 25, a minimum-samples parameter of 10, and Euclidean distance. Samples not assigned to a cluster by HDBSCAN were labelled as noise. The number of detected clusters, number and fraction of noise samples, number and fraction of cluster-assigned samples, and cluster-size distributions were recorded for each value of $\lambda_{\alpha }$.

The similarity of each clustering solution to the primary $\lambda_{\alpha }=0.20$ solution was quantified using the adjusted Rand index (ARI). Two complementary measures were calculated. First, ARI was calculated across all matched patients, including HDBSCAN noise as label −1:

(31) $${\mathrm{ARI}}_{\mathrm{all}}=\mathrm{ARI}\left(C^{\left(0.20\right)},C^{\left(\lambda_{\alpha }\right)}\right).$$

Second, ARI was recalculated after restricting the comparison to patients assigned to a non-noise cluster in both the reference and comparison solutions:

(32) $${\mathrm{ARI}}_{\mathrm{assigned}}=\mathrm{ARI}\left(C_{A}^{\left(0.20\right)},C_{A}^{\left(\lambda_{\alpha }\right)}\right),$$

where A denotes the subset of patients with cluster labels ≥0 in both solutions. This analysis was used to distinguish robustness of the continuous response-space structure from stability of any discrete cluster partition.

### 4.16. Model-Parameter Sensitivity Analysis

Sensitivity to the numerical parameterisation of the shared dynamical system was evaluated by globally scaling two parameter classes around the primary model. All non-zero regulatory interaction weights were multiplied by 0.80 or 1.20 while preserving network topology and interaction signs. In separate analyses, all reference production parameters $\alpha_{0,i}$ were multiplied by 0.80 or 1.20 while retaining the sample-specific transcriptomic modulation of each production parameter. All remaining model settings were unchanged. For each alternative parameterisation, the complete six-condition simulation panel and 42-feature behavioural fingerprints were regenerated. Robustness was assessed from rank correlations of perturbation-response features with the primary model, explained variance of PC1–PC3, correspondence between the resulting principal-component scores, the cross-perturbation association between the proliferative response to AR suppression and the EMT response to TGF-β induction, and the principal Gleason and TP53 associations. This analysis was intended as a local sensitivity assessment of the selected parameterisation rather than a formal parameter-identifiability or global uncertainty analysis.

### 4.17. Combined-Perturbation Non-Additivity Analysis

To determine whether the responses to combined perturbations deviated from the sum of their corresponding single-perturbation effects, sample-level non-additivity was evaluated for the AR suppression plus PI3K/AKT suppression and AR suppression plus TGF-β induction conditions. The analysis was performed separately for AR activity, proliferative activity, EMT activity, survival, and DDR.

For tumour sample *p*, output *m*, and a combination of perturbations $q_{1}$ and $q_{2}$, the additive expectation was defined as the sum of the two single-perturbation changes from baseline:

(33) $$\Delta F_{m,p}^{\left(\mathrm{add}\right)}=\Delta F_{m,p}^{\left(q_{1}\right)}+\Delta F_{m,p}^{\left(q_{2}\right)}.$$

The sample-level non-additivity index was then calculated as

(34) $$I_{m,p}^{\left(q_{1},q_{2}\right)}=\Delta F_{m,p}^{\left(q_{1}+q_{2}\right)}-\left[\Delta F_{m,p}^{\left(q_{1}\right)}+\Delta F_{m,p}^{\left(q_{2}\right)}\right].$$

Accordingly, I=0 indicates exact additivity within the model, whereas positive or negative values indicate deviations above or below the additive expectation, respectively. These deviations were treated as model-level non-additivity and were not interpreted as pharmacological synergy or antagonism.

For each combination–output pair, the distribution of sample-level non-additivity indices was summarised using the mean, standard deviation, median, 5th, 25th, 75th, and 95th percentiles, together with the fractions of positive, negative, and near-zero values. Departure of the median distribution from zero was assessed using a two-sided Wilcoxon signed-rank test. Benjamini–Hochberg false-discovery-rate correction was applied jointly across the 10 combination–output tests. Because these comparisons were based on deterministic model simulations rather than repeated experimental observations, the Wilcoxon tests were treated as descriptive tests of systematic departure from zero across the simulated cohort and not as measures of model or parameter uncertainty. Interpretation therefore focused primarily on the absolute and relative magnitudes of the non-additivity indices rather than on their nominal statistical significance.

To quantify the magnitude of non-additivity relative to the corresponding combined perturbation response, a descriptive relative non-additivity measure was calculated as

(35) $$R_{m}^{\left(q_{1},q_{2}\right)}=\frac{{\mathrm{median}}_{p}\left(\left|I_{m,p}^{\left(q_{1},q_{2}\right)}\right|\right)}{{\mathrm{median}}_{p}\left(\left|\Delta F_{m,p}^{\left(q_{1}+q_{2}\right)}\right|\right)}.$$

This quantity was treated as a descriptive effect-magnitude measure rather than a statistical interaction coefficient. When the median absolute combined response approached zero, the relative measure was considered numerically unstable and was not used for biological interpretation.

Finally, sample-level coordination of non-additive responses across functional outputs was explored separately for each combined perturbation using pairwise Spearman rank correlations between non-additivity indices. Benjamini–Hochberg correction was applied across the valid pairwise correlation tests.

### 4.18. Personalisation Ablation Analysis

An ablation analysis was performed to distinguish the contribution of patient-specific initial states from that of patient-specific production parameters. Two otherwise identical digital-twin configurations were compared. In the $x_{0}$-only configuration, transcriptome-derived node scores were used to define patient-specific initial states, whereas the basal production parameters were fixed at the shared reference values $\alpha_{0}$. In the primary $x_{0}+\alpha$ configuration, both the initial states and basal production parameters were patient specific, using $\lambda_{\alpha }=0.20$ and standardised node-score clipping at ±2.5. Network topology, decay parameters, simulation settings, perturbation definitions, and output calculations were identical between the two configurations. Because the $x_{0}$-only models shared all dynamical parameters, convergence towards similar terminal states was considered an expected property of this ablation configuration rather than evidence that initial-state personalisation alone should preserve steady-state heterogeneity.

For both configurations, the complete six-condition simulation panel was rerun for all 492 tumour samples and the same 42-feature behavioural fingerprints were reconstructed. To permit direct comparison of response-space amplitude, a common feature transformation was used. A standard scaler was fitted to the 42 behavioural features from the primary $x_{0}+\alpha$ model and the resulting transformation was applied unchanged to both configurations. Thus, differences in dispersion between the two models were retained rather than removed by independent within-model standardisation.

Global response-space dispersion was characterised using Euclidean distances from each sample to the cohort centroid in the commonly scaled 42-dimensional feature space. Total feature variance was additionally calculated as the sum of the sample variances of the 42 scaled features:

(36) $$V_{\mathrm{total}}=\sum_{k=1}^{42}\mathrm{Var}\left({\tilde{F}}_{k}\right).$$

Inter-patient separation was further quantified from all pairwise Euclidean distances in the same commonly scaled feature space. Their median, mean, 5th percentile, and 95th percentile were recorded for each model configuration.

Baseline steady-state heterogeneity was assessed separately for AR activity, proliferation, EMT, survival, and DDR by calculating the sample standard deviation of each unscaled baseline output across patients. Feature-level sample variances were also calculated for all 42 unscaled behavioural features in both configurations.

To compare the intrinsic dimensional structure of the two response spaces, PCA was fitted separately within each model configuration after application of the common scaling transformation. Fifteen principal components were retained, and the explained-variance ratio and cumulative explained variance were recorded for each configuration.

As a secondary descriptive analysis, HDBSCAN was independently applied to each 15-dimensional PCA representation using a minimum cluster size of 25, a minimum-samples parameter of 10, and Euclidean distance. This clustering analysis was used only to characterise the response spaces generated by the two personalisation schemes and was not treated as evidence for stable discrete patient phenotypes.

### 4.19. External Experimental Benchmark of AR-Suppression Responses

The direction of selected simulated responses to AR suppression was compared with two independent LNCaP transcriptomic perturbation datasets. GSE220097 was used as the primary experimental benchmark and compared R1881-stimulated LNCaP cells with cells exposed to R1881 plus enzalutamide for 22 h (10 control and 5 treated samples). GSE202755 provided an additional directional comparison and included DHT-stimulated LNCaP cells and cells exposed to DHT plus enzalutamide for 72h (3 control and 3 treated samples).

Experimental expression profiles were transformed as $\log_{2}\left(\mathrm{expression}+1\right)$, with duplicate gene symbols collapsed by their mean where required. Five Hallmark gene sets were used to construct experimental scores corresponding to modelled functional outputs:

- HALLMARK_ANDROGEN_RESPONSE for the AR programme,
- HALLMARK_E2F_TARGETS for proliferation,
- HALLMARK_EPITHELIAL_MESENCHYMAL_TRANSITION for EMT,
- HALLMARK_DNA_REPAIR for DDR,
- HALLMARK_APOPTOSIS for apoptosis.

For each sample, the corresponding gene-set score was calculated as the mean transformed expression of represented genes.

Differences between enzalutamide-treated and control conditions were summarised as changes in mean gene-set score and evaluated using two-sided Mann–Whitney *U* tests with rank-biserial effect sizes. The direction of each experimental change was compared with the corresponding median simulated AR-suppression response. To assess whether differences in gene coverage between the two experimental datasets affected this comparison, the analysis was repeated using only genes represented in both datasets for each gene set.

These analyses were intended as independent experimental benchmarks of response direction rather than as quantitative validation of the digital-twin model. The experimental systems were prostate-cancer cell lines rather than patient tumours and differed in androgen stimulus, exposure duration, and experimental context.

For supplementary visualisation of perturbation-response patterns across outputs with markedly different absolute response magnitudes, each output-specific median response was divided by the maximum absolute median response for that output across the five perturbation conditions. This yielded values between −1 and 1 while preserving response direction and relative magnitude within each output. The scaling was used only for visualisation; all quantitative and statistical analyses were performed using the original unscaled responses.

### 4.20. Software and Reproducibility

All computational analyses were performed in Python 3.12.15 within a dedicated Conda environment. Data processing and tabular operations were performed using NumPy and pandas; numerical integration and statistical procedures used SciPy; feature standardisation, principal-component analysis, and associated multivariate procedures used scikit-learn; and density-based clustering was performed using HDBSCAN. Matplotlib 3.10.9 was used for graphical output. Intermediate and final tabular datasets were stored in tab-separated text or Apache Parquet format as appropriate.

The computational workflow was implemented as a sequence of modular scripts covering data acquisition, sample harmonisation, transcriptomic preprocessing, Reactome pathway scoring, functional-node construction, patient-specific model parameterisation, dynamical simulation, behavioural-fingerprint generation, dimensionality reduction, clinicomolecular association analyses, static-transcriptomic comparison, tumour-purity analysis, copy-number analysis, external experimental benchmarking, sensitivity analyses, and figure generation. Patient identifiers were retained consistently across processing stages to enable deterministic linkage of transcriptomic, mutation, clinical, and simulation-derived data.

All hypothesis tests were two-sided unless otherwise specified. Where multiple related hypotheses were tested, nominal *p*-values were adjusted using the Benjamini–Hochberg false-discovery-rate procedure as described for the corresponding analysis. Statistical effect sizes and descriptive measures were reported alongside significance tests where applicable. No missing clinical, mutation, copy-number, or tumour-purity annotations were imputed; analyses requiring a particular post-hoc annotation were restricted to samples for which that annotation was available. This treatment of annotation-level missingness was distinct from the zero filling used during construction of the gene-by-sample RNA-sequencing count matrix, where absent entries introduced during matrix alignment were assigned zero before CPM normalisation.

Reactome pathway definitions were obtained from the official Reactome current-release distribution ReactomePathways.gmt.zip), downloaded on 1 February 2026. The extracted pathway definition file (ReactomePathways.gmt) was retained with its SHA-256 checksum (a34f43fd763a6800a9a94310c416ae511e84724d87a3c255f35d13764d12bebb) to enable verification of the exact pathway resource used in the analysis. As the data were retrieved from the Reactome current-release directory, the numerical release identifier was not recorded in the original download metadata.

The complete analysis code and environment specification are publicly available through Zenodo.

## 5. Conclusions

These findings support functional response modelling as a complement to conventional molecular profiling. Transcriptome-informed digital twins provide a framework for characterising how patient-specific molecular states translate into differences in simulated responses to defined pathway perturbations. Rather than identifying stable discrete response phenotypes, the analysis revealed a continuous functional response landscape associated with tumour grade and selected genomic characteristics. The association between the dominant response axis and Gleason score persisted after adjustment for estimated tumour purity, although this does not establish independence from other clinical or molecular factors. The dominant response axis largely preserved the major variation already present in the transcriptomic inputs, while dynamical modelling provided an explicit framework for translating these molecular states into baseline and counterfactual perturbation responses.

This framework is intended for pathway-level functional response modelling rather than for treatment prediction. The present model should therefore be regarded as a mechanistic, hypothesis-generating re-expression of molecular heterogeneity rather than as a clinically validated predictive digital twin. Future versions of the framework will require longitudinal molecular data, experimentally calibrated perturbations, independent patient cohorts, and explicit uncertainty quantification. Integration of pharmacokinetic and pharmacodynamic information would additionally be required before extending the framework towards treatment-specific prediction. The framework may be adaptable to other cancer types and to additional classes of molecular data from which tumour-specific perturbation responses can be modelled.

## Figures and Tables

**Table 1:** Reference parameters and regulatory interactions of the eight-node digital-twin model. Positive regulatory weights indicate activation, whereas negative weights indicate inhibition. All regulatory interactions not listed in the table were set to zero.

Node-specific reference parameters			
**Functional node**		$\alpha_{0,i}$	$\delta_{i}$
AR programme		0.60	0.60
PI3K/AKT		0.50	0.60
MAPK/ERK		0.35	0.60
TGF-β		0.35	0.60
EMT/ECM/invasion		0.30	0.60
Cell-cycle/proliferation		0.45	0.60
Apoptosis/TP53		0.35	0.60
DDR		0.35	0.60
Directed regulatory interactions			
**Source node**	**Target node**	$W_{ij}$	
AR programme	Cell-cycle/proliferation	+0.60	
AR programme	EMT/ECM/invasion	−0.30	
AR programme	PI3K/AKT	−0.10	
PI3K/AKT	Cell-cycle/proliferation	+0.50	
PI3K/AKT	Apoptosis/TP53	−0.40	
PI3K/AKT	AR programme	−0.20	
MAPK/ERK	Cell-cycle/proliferation	+0.35	
MAPK/ERK	EMT/ECM/invasion	+0.25	
TGF-β	EMT/ECM/invasion	+0.70	
TGF-β	Cell-cycle/proliferation	−0.20	
EMT/ECM/invasion	Apoptosis/TP53	−0.20	
EMT/ECM/invasion	AR programme	−0.30	
DDR	Apoptosis/TP53	+0.60	
Apoptosis/TP53	Cell-cycle/proliferation	−0.50	

$\alpha_{0,i}$, reference basal production parameter for node *i*; $\delta_{i}$, first-order decay parameter; $W_{ij}$, directed regulatory weight from source node *j* to target node *i*; AR, androgen receptor; PI3K/AKT, phosphoinositide 3-kinase/protein kinase B; MAPK/ERK, mitogen-activated protein kinase/extracellular signal-regulated kinase; TGF-β, transforming growth factor beta; EMT, epithelial–mesenchymal transition; ECM, extracellular matrix; DDR, DNA-damage response.

**Table 2:** In silico perturbation conditions applied to the sample-specific digital twins. Perturbation strengths were defined as functions of the corresponding sample-specific initial node states, $x_{i}\left(0\right)$. For combined perturbations, the respective single-perturbation factors were applied simultaneously without an additional interaction term.

Condition	Target Node(s)	Perturbation Factor	Range
Baseline	None	$e_{i}=1$	1.00
AR suppression	AR programme	$e_{\mathrm{AR}}=0.55-0.40x_{\mathrm{AR}}\left(0\right)$	0.15–0.55
PI3K suppression	PI3K/AKT	*e*_PI3K_ = 0.70 − 0.35*x*_PI3K_(0)	0.35–0.70
TGF-β induction	TGF-β	*e*_TGF*β*_ = 1.20 + 0.80[1 − *x*_TGF*β*_(0)]	1.20–2.00
AR + PI3K suppression	AR programme; PI3K/AKT	$e_{\mathrm{AR}}$ and *e*_PI3K_ applied simultaneously	As above
AR suppression + TGF-β induction	AR programme; TGF-β	$e_{\mathrm{AR}}$ and *e*_TGF*β*_ applied simultaneously	As above

Note: $e_{i}$, multiplicative perturbation factor applied to node *i*; $x_{i}\left(0\right)$, sample-specific initial state of node *i*. Values of $e_{i}<1$ represent suppression, whereas values of $e_{i}>1$ represent induction. All nodes not targeted by a given perturbation retained $e_{i}=1$. For combined conditions, the corresponding sample-specific perturbation factors were applied simultaneously.

## Data Availability

The datasets processed, software and output tables/data for this study can be found in the Zenodo under the DOI: 10.5281/zenodo.23659602 (accessed on 26 October 2026).

## Supplementary Materials

The following supporting information can be downloaded at https://zenodo.org/records/23659835 (accessed 26 October 2026).

## Appendix A. Distribution of Transcriptome-Derived Functional-Node Scores

**Table A1:** Distribution of transcriptome-derived functional-node scores across the TCGA-PRAD cohort (n=492).

Functional Node	*n*	Mean	SD	Min	Q1	Median	Q3	Max
AR programme	492	−0.0022	0.2468	−1.3519	−0.0983	0.0494	0.1516	0.4701
PI3K/AKT	492	−0.0015	0.3264	−1.7313	−0.1381	0.0584	0.2065	0.6865
MAPK/ERK	492	−0.0020	0.3135	−1.7820	−0.1151	0.0646	0.1924	0.6099
TGF-β	492	−0.0043	0.3886	−2.2960	−0.1648	0.0803	0.2564	0.7473
EMT/ECM/invasion	492	−0.0014	0.3964	−1.5789	−0.2157	0.0449	0.2857	0.9289
Cell cycle/proliferation	492	−0.0043	0.3746	−2.3356	−0.1943	−0.0092	0.2046	1.3241
Apoptosis/TP53	492	−0.0034	0.3104	−2.0261	−0.1252	0.0461	0.1830	0.6877
DDR	492	−0.0022	0.4108	−2.2483	−0.2016	0.0260	0.2130	1.6405

Note: Values represent transcriptome-derived functional-node scores calculated by median aggregation of the corresponding Reactome pathway activity scores. Q1 and Q3 denote the first and third quartiles, respectively; SD, standard deviation; DDR, DNA-damage response.
